# Quantitative trait loci and candidate genes associated with freezing tolerance of winter triticale (× *Triticosecale* Wittmack)

**DOI:** 10.1007/s13353-021-00660-1

**Published:** 2021-09-07

**Authors:** I. Wąsek, M. Dyda, G. Gołębiowska, M. Tyrka, M. Rapacz, M. Szechyńska-Hebda, M. Wędzony

**Affiliations:** 1 Institute of Biology, Pedagogical University of Cracow, Podchorążych 2, 30-084 Kraków, Poland; 2 Department of Biotechnology and Bioinformatics, Faculty of Chemistry, Rzeszow University of Technology, Powstańców Warszawy 6, 35-959 Rzeszow, Poland; 3 Department of Plant Breeding, Physiology and Seed Science, University of Agriculture in Kraków, Podłużna 3, 30-239 Krakow, Poland; 4 Plant Breeding and Acclimatization Institute, National Research Institute, 05-870 Radzików, Błonie Poland; 5 The Franciszek Górski Institute of Plant Physiology, Polish Academy of Sciences, Krakow, Poland

**Keywords:** Freezing tolerance, Plant acclimation, Genetic map, QTL, Chlorophyll fluorescence, Transmembrane, Proteins, Regulation of gene expression, Cereals

## Abstract

Freezing tolerance of triticale is a major trait contributing to its winter hardiness. The identification of genomic regions — quantitative trait loci (QTL) and molecular markers associated with freezing tolerance in winter hexaploid triticale — was the aim of this study. For that purpose, a new genetic linkage map was developed for the population of 92 doubled haploid lines derived from ‘Hewo’ × ‘Magnat’ F_1_ hybrid. Those lines, together with parents were subjected to freezing tolerance test three times during two winter seasons. Plants were grown and cold-hardened under natural fall/winter conditions and then subjected to freezing in controlled conditions. Freezing tolerance was assessed as the plants recovery (REC), the electrolyte leakage (EL) from leaves and chlorophyll fluorescence parameters (JIP) after freezing. Three consistent QTL for several fluorescence parameters, electrolyte leakage, and the percentage of the survived plants were identified with composite interval mapping (CIM) and single marker analysis (SMA). The first locus *Qfr.hm-7A.1* explained 9% of variation of both electrolyte leakage and plants recovery after freezing. Two QTL explaining up to 12% of variation in plants recovery and shared by selected chlorophyll fluorescence parameters were found on 4R and 5R chromosomes. Finally, main locus *Qchl.hm-5A.1* was detected for chlorophyll fluorescence parameters that explained up to 19.6% of phenotypic variation. The co-located QTL on chromosomes 7A.1, 4R and 5R, clearly indicated physiological and genetic relationship of the plant survival after freezing with the ability to maintain optimal photochemical activity of the photosystem II and preservation of the cell membranes integrity. The genes located in silico within the identified QTL include those encoding BTR1-like protein, transmembrane helix proteins like potassium channel, and phosphoric ester hydrolase involved in response to osmotic stress as well as proteins involved in the regulation of the gene expression, chloroplast RNA processing, and pyrimidine salvage pathway. Additionally, our results confirm that the JIP test is a valuable tool to evaluate freezing tolerance of triticale under unstable winter environments.

**Supplementary Information:**

The online version contains supplementary material available at 10.1007/s13353-021-00660-1.

## Introduction

Freezing is one of the environmental stresses which can constrain agricultural production of winter crops; therefore, increasing freezing tolerance is still important for breeding programs in cold and temperate climates. Many plant species including hexaploid winter triticale (x *Triticosecale* Wittm.) have developed a specific adaptive response which allows to stand low and freezing temperatures. During cold acclimation process, plants can increase their freezing tolerance after an exposition to low but non-freezing temperatures (Levitt 1980; Winfield et al. 2010; Janeczko et al. 2019). In natural conditions, this process is initiated by the decreasing temperature in late autumn which is complex phenomenon associated with many molecular, biochemical and physiological changes (Salinas 2002; Kaplan et al. 2004; Catala and Salinas 2008; Burbulis et al. 2011).

It has been previously reported that low temperature alters gene expression of a large number of genes encoding proteins could potentially contribute to plant freezing tolerance (Thomashow 1998; Kovi and Ergon, 2016). Certain transcriptional responses are common for most of plant species such as induced expression of vernalization genes, *CBF* genes, and *COR* genes (Knox et al. 2008, 2010). Vernalization gene group contain a *VRN1*, *VRN2* and *VRN3* genes, whereas *CBF* gene group includes a set of tandemly duplicated *CBF* (C-repeat Binding Factors) transcription factors at the *FR2* (Frost Resistance 2) locus (Galiba et al. 2009). Furthermore, level of *VRN1* transcript increase during exposure to low temperatures (Kobayashi et al. 2005) and it can generate conversion from the vegetative to reproductive plant growth stage (Stockinger et al. 2007). This transition is also associated with the suppressed induction of *CBF* group of genes in response to cold which results in reduced frost tolerance (Kobayashi et al. 2005; Dhillon et al. 2010; Zhu et al., 2014). Additionally, *CBF* similarly to *COR* genes enhance photosynthetic capacity which was reported in *A. thaliana* and *B. napus* photosynthetic capacity and freezing tolerance in response to temperature conditions (Kurepin et al. 2013). Furthermore, accumulation of proteins encoded by *COR* genes can lead to cell membrane stability under freezing conditions (Dong et al. 2002). 

The major group of genes associated with cereal freezing tolerance have been reported and identified on the long arms of homeologous group 5 (Roberts 1990; Sutka 1994; Sutka and Snape 1989; Veisz and Sutka 1993; Kocsy et al., 2010). Also, Danyluk et al. (1994) discovered that *wCOR410* and *wCOR719* genes expression was regulated by factors located on chromosome 5A. Another locus, *Fr-A1* on long arm of chromosome 5A was mapped in a close position to the previously described vernalization gene *Vrn*-A1 (Galiba et al. 1995). The presence of loci controlling expression of *cor14b* gene was identified on the long arm of 5A in wheat by Vágújfalvi et al. (2000, 2003). Additionally, wheat chromosome 5D was proved to be involved in the regulation of freezing tolerance (Snape et al. 1997).

The measurement of chlorophyll fluorescence parameters proved to be the good method to evaluate the freezing damage of plants reflecting freezing damages of photosynthetic apparatus (Rizza et al. 2001; Rapacz et al. 2011, 2015b). Also the measurements of electrolyte leakage in freeze-damaged leaves are commonly used for estimation of freezing injury as freeze–thaw damages results in disintegration of plasma membranes (Dexter et al. 1932). However, the results of chlorophyll fluorescence measurements, electrolyte leakage test, and plant survival count may give sometimes distinct results depending on environmental conditions (Rapacz et al. 2015a).

Thus, we hypothesized that different effects of freezing on plants may be, at least partially controlled by different genes. In our study we decided to use quantitative trait loci (QTL) mapping technology performed on a newly developed genetic map for triticale to determine the number and to map position of loci of different measures of freezing tolerance such as chlorophyll fluorescence parameters (JIP test) after freezing, membrane integrity affected by freezing, and plant recovery after freezing tests field-laboratory freezing tests. It is known that interactions among loci or between genes/QTL and environment make a substantial contribution to variation in complex traits (Gupta et al. 2007). Additionally, we aimed to identify candidate genes located in genome regions associated with analyzed traits. Doubled haploid mapping population used in our study brought us a unique opportunity to perform all of the experiments to ensure sound quality of the results.

## Materials and methods

### Plant material

The mapping population consisting of 92 doubled haploid lines was derived from *F*_1_ generation of a cross between two triticale cultivars: cv. ‘Hewo’ used as a female parent (Strzelce Plant Breeding Ltd.) and cv. ‘Magnat’ as the pollen parent (Danko Plant Breeding Ltd.). Both parental cultivars differed in tolerance to *Microdochium nivale* infection (Gołębiowska and Wędzony 2009), as well as in freezing tolerance in pre-tests (unpublished data). The DH ‘Hewo x Magnat’ lines population was developed by the androgenesis in the anther culture according to the method described by Wędzony (2003). The obtained DH lines were numbered from 1 to 92 in relation to the tests results of the degree of *Microdochium nivale* tolerance. This numbering has been kept for all experiments.

### Genetic linkage map

The genomic DNA was isolated from young triticale leaves according to method described by Tyrka et al. (2011). Total DNA purity and integrity were tested on the agarose gels while its quantity was measured using UV–Vis Q500 spectrophotometer (Quawell, San Jose, USA).

Samples were genotyped in the Diversity Arrays Technology Pty Ltd (DArT P/L, Australia, www.diversityarrays.com) to detect different types of DNA variation (single nucleotide polymorphism, indel, and methylation) and to search for diagnostic markers. Using JoinMap 4 (Van Ooijen 2006) software, the segregation data were analyzed to group all markers with LOD value > 3.0. Afterwards, markers within these groups were ordered using the RECORD program (Van Os et al. 2005). The marker order was used to sort all markers within linkage groups and graphical genotypes were examined in Excel 2003. At this step, singletons were replaced by missing values in the dataset and calculations were repeated until no singletons were found (through three rounds). The distance between loci was calculated with the Kosambi function (Kosambi 1944). Finally, the final map has consisted of 680 DArT markers. Nomenclature of markers was synchronized with previously published map for partially overlapping set of lines (Tyrka et al. 2015). Consensus triticale DArT map was used for group identification and orientation (Tyrka et al. 2018).

Simple sequence repeat (SSR) analyses comprised 56 markers that were polymorphic between the two parental lines. The selected 37 mapped SSRs included the following: 20 *wms*: *gwm46, gwm95, gwm126, gwm130, gwm136, gwm146, gwm149, gwm164, gwm169, gwm181, gwm275, gwm332, gwm335, gwm339, gwm368, gwm375, gwm388, gwm495, gwm499,* and *gwm566* (Röder et al. 1998); 7 *scm*: *scm101, scm120, scm138, scm180, scm268, scm28,* and *scm304* (Saal and Wricke 1999; Hackauf and Wehling 2002); 7 *wmc*: *wmc168, wmc219, wmc262, wmc289, wmc327, wmc434,* and *wmc537*; and 3 other SSR markers: *barc182, gdm109,* and *gdm147* (Pestsova et al. 2000; Somers et al. 2004). Four primer pairs (*barc182, scm304, wmc168,* and *wmc327*) revealed simultaneously two different loci. Redundant markers were shortlisted in final genetic map, and single representative markers with the lowest number of missing data were left to represent the bin with a total number of binned markers given in brackets.

### Freezing tolerance assessment with the field-laboratory method

All presented tests took place in Kraków, Poland (N 50.069014, E 19.845528), according to the Koch and Lehman (1966) method with minor modifications, in three independent experiments with three replicates each, in the autumn/winter seasons. Three replicates were performed in a randomized complete block design in order to limit the error resulting from the marginal position of an individual genotype in the planting plastic boxes (30 cm × 38 cm × 9 cm). Kernels were sown in 13 rows of 10 kernels/genotype/box. One row per each parental line was sown in randomized positions in every box as a control. Total of 8 planting boxes/replicate, 24 boxes per each experiment were planted and analyzed.

**Fig. 1:**
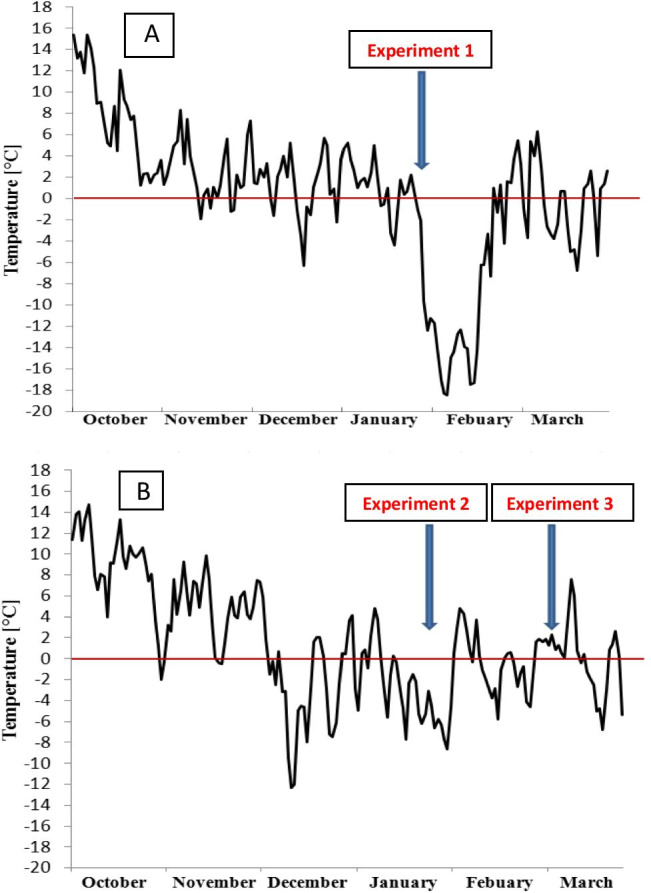
Daily temperatures (means) measured in the open-air vegetation chamber in autumn/winter seasons 2012/2013 (**A**) and 2013/2014 (**B**). The dates of plant transfer to the freezing chamber (the start of the freezing test), leaves sampling for chlorophyll fluorescence measurements (JIP test), and electrolyte leakage measurements (on **B**) are indicated by arrows: 26 January 2012 (**A**), 21 January 2013, and 4 March 2013 (**B**). The temperature was monitored with an electronic weather station (Ogimet)

Plants were grown and cold-hardened under natural fall/winter conditions in the open-air vegetation chamber. The temperature was monitored with an electronic weather station WS-3600–11, Technoline, Berlin, Germany, and the mean values of daily temperatures are presented in Fig. 1. On the days indicated by arrows (Fig. 1), the exact number of growing seedlings was counted in each row, and the boxes with plants were moved to the freezing chamber where they were subjected to the cycle consisting of one day-long growth in − 2 °C, followed by gradual temperature decrease (3 °C/h) down to − 15 °C, and then stable for 6 h. Later, the temperature was increased at the rate 3 °C/h to + 2 °C. After reaching + 2 °C, the boxes were transferred to an unheated glasshouse maintained at 10–15 °C, and the plants were cut 2 cm above the soil level. After 3 weeks, the number of surviving (regrowing) plants was established and the plant survival was expressed as a percentage of the survived plants from initially growing plants.

### Chlorophyll a fluorescence measurements

Parameters of induction kinetics of chlorophyll *a* fluorescence in dark-adapted leaves (JIP test) were measured on the middle section of the second fully expanded detached leaves with HandyPEA fluorimeter (Hansatech Kings Lynn, UK). On the days indicated by arrows in Fig. 1 (experiments 1–3), before boxes with plants were put into the freezing chamber to perform freezing tests, 10–12 leaves were randomly collected from each DH line, approximately the same number of 3–4 leaves/DH line from each of 3 replications. Since parental lines were present as a control in every box, parental samples consisted of approximately 26 leaves per each of 3 replications. Leaves were packed into polyethylene bags with a string closure and frozen in − 15 °C for 6 h with the protocol described by Rapacz et al. (2015a). Then, the leaves were brought to room temperature and dark-adapted before measurements for 15 min in a leaf clip (Hansatech, Kings Lynn, UK). Chlorophyll *a* transient fluorescence was measured according to Rapacz (2007) with a light pulse intensity of 3500 μmol m^−2^ s^−1^, and the pulse duration for 0.7 s with the fixed gain (1 ×). The following parameters of JIP test were calculated and described as in Rapacz (2007): absorbed energy flux per leaf cross-section (CS) and the single, active PSII reaction center (RC) (ABS/CS, ABS/RC respectively); trapped energy flux in PSII reaction centers per leaf cross-section and the single, active PSII reaction center (Tr_0_/CS, Tr_0_/RC, respectively); the energy flux for electron transport per leaf cross-section and the single, active PSII reaction center (ET_0_/CS, ET_0_/RC, respectively); dissipation of energy in PSII reaction centers per leaf cross-section and the single, active PSII reaction center (DI_0_/CS, DI_0_/RC, respectively); yield of the energy trapping in PSII (*F*_v_/*F*_m_); performance indexes of PSII (PI) normalized for minimal and maximal densities of active reaction centers per leaf cross-section (PI_CS0_ and PI_CSm,_ respectively); minimal and maximal densities of active reaction centers per leaf cross-section (RC/CS_0_ and RC/CS_m_, respectively); and the quantum yield of electron transport (φEo) and the efficiency of the electron transfer from QA- to QB (ψo).

### Electrolyte leakage measurements

The electrolyte leakage considered as the test of plasma membrane damages was performed in accordance with Flint et al. (1967), two times during winter 2013/2014 (dates indicated by arrows on Fig. 1B, experiments 2 and 3). In each series, measurements were made in 20 biological replicates for each genotype (1 leaf from different plant = 1 replicate). Each leaf was placed separately in a 20-cm^3^ plastic tube filled with 5 cm^3^ of deionized water. The material was frozen for 6 h at − 15 °C with the protocol described for chlorophyll fluorescence studies. After removing from the freezing chamber, 10 cm^3^ of deionized water was added to each tube and then the samples were shaken (ROTH, Linegal Chemicals Sp. z o.o) at room temperature for 24 h. Then, conductivity measurements (EL1) were performed in each tube using a conductivity meter type OK 102/1 (Radelkis). Probes were then frozen in liquid nitrogen for 2 min and again shaken at room temperature for 24 h before the second electrical conductivity measurement (EL2). The EL % was calculated as EL = (EL1/EL2) × 100%, where EL1 = primary electrolyte leakage after − 15 °C treatment and EL2 = total electrolyte leakage after freezing in liquid nitrogen.

### Statistical analysis

All the data were analyzed with Statistica 13.0 PL software (Statsoft, Tulsa, OK, USA). Distribution of the data was checked using histograms and accompanied with a Shapiro–Wilk test. For JIP test parameters, one-dimensional variance analysis was performed. Linear correlation coefficients (Pearson’s) were calculated for each of three experiments separately on the basis of mean value of replicates per genotype: (1) between percentage of survived plants and every parameter of fluorescence and (2) between the fluorescence parameters and electrolyte leakage (experiments 2 and 3). The regression line was presented with a 95% coefficient interval.

### Quantitative trait loci (QTL) identification

To identify QTL regions associated with the analyzed traits, single marker analysis (SMA) and composite interval mapping (CIM) methods were calculated with the Windows QTLCartographer software version 2.5 (Wang et al. 2012). SMA analysis fits the data to the simple linear regression model while CIM method in turn, determines the linkage between QTL and markers limiting the designated interval on the chromosome map. The threshold logarithms of the odds (LOD) scores were calculated based on 1000 permutations and 1 cM walk speed. QTL was accepted for LOD scores higher than 2.5. The percentage of phenotypic variation was calculated with a single factor regression (*R*^2^). Favorable alleles in each QTL region were selected on the basis of the additive effect (Add), where negative additive effect referred to cv. ‘Magnat’ and positive referred to cv. ‘Hewo’. The CIM and SMA analyses were performed separately for each experiment. Results of QTL analysis were visualized using CorelDRAW9 software.

The label of each identified QTL region was created from the short name of each parameter (ex., Qrec for recovery, survival; Qel for electrolyte leakage). For loci explained more than one trait, the general name was given (ex., Qfr for loci co-located for recovery, survival, and electrolyte leakage; Qchl for loci containing analyzed chlorophyll *a* fluorescence parameters) Hewo × Magnat (hm), chromosome names (wheat A and B group, rye group R), and QTL number on the chromosome (1–4).

### The in silico location of genes within the QTL

Within the localized QTL, candidate genes associated with the analyzed traits were identified in silico according to Karbarz et al. (2020) with modifications. Sequences corresponding to the wheat, rye and triticale DArT clones of the flanking and maximal LOD peak markers of the significant QTLs were downloaded from the Diversity Arrays Technology webpage (https://www.diversityarrays.com/technology-and-resources/sequences/). Then, DArT sequences were used to query the IWGSC RefSeq 1.0 wheat genome for physical mapping using the BLAST tool in the Unité de Recherche Génomique URGI database (https://urgi.versailles.inra.fr). Subsequently, DArT sequences were used to query all available wheat and rye genome collections for physical mapping using the BLAST tool of GrainGenes Blast Service beta (10.1093/molbev/msz185). Genes localized on target physical wheat and rye regions were retrieved and annotated with the use of BLAST® (https://blast.ncbi.nlm.nih.gov/Blast.cgi); the sequences producing significant alignments and the highest query cover were selected. Next, the function of candidate genes was deduced from the UniProt database.

## Results

### Genetic map of the DH ‘Hewo’ x ‘Magnat’ population with SSR and DArT markers

A set of 92 DH lines derived from F_1_ triticale plants that originated from a cross between cv. ‘Hewo’ and cv. ‘Magnat’ were used to create a new and unique genetic linkage map. Upon the multiple-mapping approaches, a total of 41 SSR and 680 diversity array technology (DArT) markers were ordered into 22 linkage groups assigned to the A, B, and R genomes (Table 1; Fig. S1 A, B, R; Table S1). The mapped markers with common segregation pattern were binned. Markers representing bins were referred as ‘unique’ while number of all markers in a bin was treated as ‘total’ (Table 1). Additionally, three chromosomes, 7A, 2B, and 3B, were represented by double linkage groups. All mapped DArT markers belonged to three groups, *rPt*, *tPt*, and *wPt*, that were developed respectively from rye, triticale, and wheat. Additionally, during the map construction a small number of markers were eliminated, mainly owing to a high percentage of missing data or a lack of linkage with established markers clusters at LOD value of 2.0. Finally, all remaining markers have covered a total of 1367.7 cM with a mean distance between two markers of 4.7 cM. Microsatellite markers allowed assignment of linkage groups to chromosomes of the A and B genomes of wheat and to five chromosomes of rye (1R, 3R, 4R, 5R, and 6R) (Table 1). Comparative analyses with triticale maps (Tyrka et al. 2011, 2015) and additional information on distribution of wheat DArT markers (series *wPt*) were provided by Diversity Arrays Technology Pty Ltd. and validated the assignment of linkage groups to rye chromosomes. The order of DArT and SSR markers that were used to develop this linkage map are presented in Supplementary Fig. S1 A, B and R and Supplementary Table S1. Details on the distribution of SSR and DArT markers across triticale genomes of Hewo x Magnat population showed the highest saturation of R genome with unique markers, whereas lower densities were identified for the A and B genomes. R genome was covered by 326 markers with total length of 290.4 cM and a mean distance between two unique markers of 2.7 cM, whereas 140 and 255 markers were distributed within genome A and B respectively, with total length of 460.8 and 616.5 cM and density 6.4 and 5.5 cM (Table 1; Table S1).

**Table 1:** Genetic linkage map developed for ‘Hewo × Magnat’ DH mapping population. The mapped markers with common segregation pattern were binned. Markers representing bins were referred as ‘unique’ while number of all markers in a bin was treated as ‘total’. The numbers in bold indicate the length of the entire map and the total number of markers

Genome	Linkage group	Lenght (cM)	Marker nos		
			Total	Unique	Density
A	1A	54.3	24	17	3.4
	2A	45.0	10	6	9.0
	3A	43.9	10	3	22.0
	4A	76.1	34	13	6.3
	5A	78.1	12	8	11.2
	6A	48.3	8	4	16.1
	7A.1	61.8	25	11	5.6
	7A.2	53.3	17	10	5.9
Total genome A		460.8	140	72	6.4
B	1B	93.3	55	21	4.7
	2B.1	65.1	16	10	7.2
	2B.2	48.6	16	8	6.9
	3B.1	22.9	14	8	3.3
	3B.2	115.1	38	18	6.8
	4B	42.5	12	7	7.1
	5B	41.9	13	8	6.0
	6B	97.6	42	16	6.5
	7B	89.5	49	17	5.6
Total genome B		616.5	255	113	5.5
R	1R	17.5	20	5	4.4
	3R	37.5	31	7	6.3
	4R	71.6	82	17	4.2
	5R	36.6	66	19	2.0
	6R	127.2	127	58	2.2
Total genome R		290.4	326	106	2.7
Total 22		**1367.7**	**721**	**291**	**4.7**

### Freezing tolerance of plants cold acclimated under field conditions

From October to January of winter 2012/2013, plants of the experiment 1 grew under an average temperature about + 4 °C, mostly above 0 °C with few days decrease below (minimum in the average daily temperature to − 6.3 °C in December) (Fig. 1A). The winter 2013/2014 was more severe. Plants of experiment 2 grew under large fluctuations of the average temperature which was mostly above 0 °C in October and November, but below 0 °C in December and January, with minimum − 12.3 °C (Fig. 1B). The period with an average temperature of approximately − 2 °C started in December and continued until March, when temperature raised to about + 2 °C just before the experiment 3 measurements were performed (Fig. 1B).

### The results of the freezing tolerance testing

**Fig. 2:**
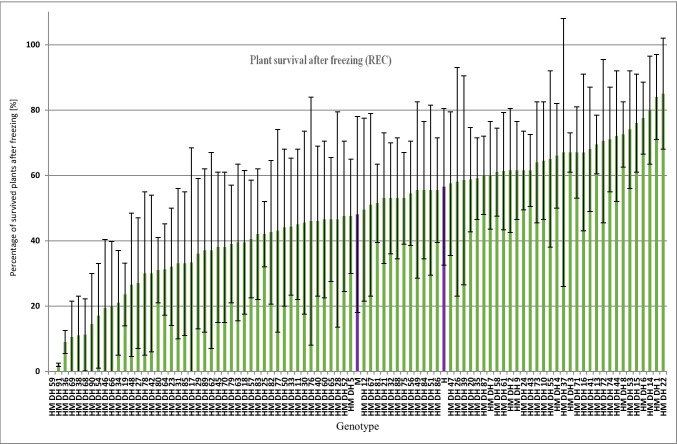
Mean variability (from three freezing tests) of the percentage of survived plants in Hewo × Magnat DH population. Violet bars represents parental cultivars Magnat (M) and Hewo (H); green bars represents individual DH lines. Error bars represent the value of standard deviation

The mean percentage of survived plants from three independent freezing test performed during winter 2012/2013 and 2013/2014 varied from 0 to 85% (Fig. 2). Transgressive segregation of plant recovery was observed in the evaluated population of DH lines. The minimal (0%) percentage of survived plants was observed for HM DH 59 line and the maximal for HM DH 22 (85%) line. Relatively high standard deviation for most DH lines clearly indicates that plants recovery strongly depend on weather conditions. Parent Hewo revealed higher percentage of survived plants after freezing (56%) than parent Magnat (48.5%).

### PSII photochemical efficiency and membrane stability after freezing

Changes in photochemical efficiency under different stresses can be expressed by the chlorophyll *a* fluorescence parameters (Kurepin et al. 2013; Rapacz 2007). In our study, the photosynthesis efficiency during cold hardening of triticale plants was evaluated based on the maximum quantum yield of photosystem II (PSII) (*F*_v_/*F*_m_), the overall performance indexes of PSII (PI) and particular JIP test parameters which were recommended by Rapacz et al. (2015a) as a good tools to evaluate freezing tolerance (RC/CS_m_, RC/CS_0_, TR_0_/CS, and ET_0_/CS). For all evaluated chlorophyll fluorescence parameters transgressive segregation was observed in our mapping population. The values of each parameter were different and dependent (Table 2, Figure S2). Relatively high standard deviation of chlorophyll fluorescence parameters for parents may result from the high impact of various weather conditions. In general, the photosynthetic apparatus for Hewo was more active and functioned better than that for Magnat, resulting in significantly higher ABS/CS (characterizing light energy absorption), RC/CS_m_ (number of active reaction centers). *F*_v_/*F*_m_ (maximum quantum yield of PSII) and PI (overall performance index of PSII photochemistry) also had trends for higher means in Hewo, though similar amounts of energy used for electron transport and amount of excitation energy trapped in PSII reaction centers (ET_0_/CS and TR_0_/CS, respectively) were for both parents. However, the dissipation of energy in PSII reaction centers (DI_0_/CS and DI_0_/RC) was higher for Magnat than Hewo.

**Table 2:** Phenotypic performance for traits related to chlorophyll a fluorescence parameters and electrolyte leakage (EL) from leaves of doubled haploid lines and their parents

Traits	Parents (mean ± SD)		DH lines		
	Hewo	Magnat	Mean ± SD	Min	Max
*F*_*v*_*/F*_*m*_	0.71 ± 0.45	0.63 ± 0.36	0.66 ± 0.05	0.54	0.77
PI	0.89 ± 0.31	0.69 ± 0.34	0.66 ± 0.13	0.32	0.95
*ψ*_o_	0.39 ± 0.12	0.38 ± 0.11	0.35 ± 0.03	0.29	0.44
φE_o_	0.28 ± 0.13	0.28 ± 0.13	0.25 ± 0.03	0.19	0.34
PI_CSo_	324.48 ± 143.21	300.42 ± 122.62	287.98 ± 54.53	142.91	425.80
PI_CSm_	1458.53 ± 426.89	1384.42 ± 512.49	1314.71 ± 319.48	525.43	2281.35
ABS/CS	432.54 ± 3.20	412.11 ± 16.77*	444.36 ± 31.73	348.74	547.45
TR_o_/CS	295.09 ± 117.95	291.41 ± 59.38	293.32 ± 31.66	223.70	364.88
ET_o_/CS	120.06 ± 58.22	115.08 ± 55.24	113.21 ± 15.14	72.19	157.70
DI_o_/CS	109.82 ± 33.17	148.65 ± 70.08*	151.45 ± 24.27	101.80	230.45
ABS/RC	5.20 ± 4.17	6.03 ± 5.53	6.27 ± 3.19	2.78	18.05
ET_o_/RC	0.87 ± 0.24	0.83 ± 0.25	0.80 ± 0.06	0.58	1.00
TR_o_/RC	2.31 ± 0.15	2.25 ± 0.16	2.34 ± 0.13	1.88	2.61
DI_o_/RC	0.77 ± 0.16	1.81 ± 0.19*	1.37 ± 0.52	0.68	3.26
RC/CS_o_	131.85 ± 39.94	127.45 ± 38.72	129.02 ± 13.61	102.28	158.47
RC/CS_m_	560.25 ± 239.73	526.87 ± 240.07*	521.11 ± 69.45	394.86	707.95
EL	69.51 ± 9.61	77.05 ± 15.54	59.12 ± 21.53	18.98	90.02

^*^Significance levels between parents ≤ 0.05

The degree of damage to cell membranes under frost was determined by the measurement of electrolyte leakage (EL) from leaf tissues. The statistically significant differences between parents were not observed. The value of the electrolyte leakage for parental cvs.: Hewo and Magnat was 69% and 77%, respectively. The DH lines differed from parental values of electrolyte leakage. The transgression segregation was clearly highlighted: 65% of the DH lines from the population were characterized by a lower statistically significant EL in relation to both Magnat and Hewo parental cultivars. In contrast, 11% of the DH lines showed a higher statistically significant EL value in relation to both parents. The min and max values of EL was 19% and 90%, respectively (Table 2).

### Correlation between plant recovery, electrolyte leakage and chlorophyll a fluorescence parameters

A relationship was established between the average percentage of survived plants from three independent experiments and the mean values of the electrolyte leakage and JIP parameters. For that purpose, the Pearson correlation coefficient (*r*) was calculated between the individual parameters (Table 3). There was no statistically significant correlation between plant recovery and electrolyte leakage. In contrast, a relatively high positive correlation (0.34 to 0.62) was found between plant recovery (REC) and JIP parameters: *F*_v_/*F*_m_, *Ψ*_0_, φE_0_, PI, PI_CSo_, PI_CSm_, ABS/CS, TR_0_/CS, ET_0_/CS, ET_0_/RC, RC/CS_0_, RC/CS_m_, and negative statistically significant correlation (− 0.32 to − 0.35) for ABS/RC, DI_0_/RC, and DI_0_/CS. The correlation between freezing tolerance and TR_0_/RC was not statistical significant (Table 3, Fig. 2S).

**Table 3:** Correlation matrix using phenotypic mean values for recovery (REC), electrolyte leakage (EL) from leave and chlorophyll a fluorescence parameters. The parameters of JIP test were calculated and described as in Rapacz (2007): absorbed energy flux per leaf cross-section (CS) and the single, active PSII reaction center (RC) (ABS/CS, ABS/RC respectively); trapped energy flux in PSII reaction centers per leaf cross-section and the single, active PSII reaction center (Tr_0_/CS, Tr_0_/RC, respectively); the energy flux for electron transport per leaf cross-section and the single, active PSII reaction center (ET_0_/CS, ET_0_/RC, respectively); dissipation of energy in PSII reaction centers per leaf cross-section and the single, active PSII reaction center (DI_0_/CS, DI_0_/RC, respectively); yield of the energy trapping in PSII (*F*_v_/*F*_m_); performance indexes of PSII (PI) normalized for minimal and maximal densities of active reaction centers per leaf cross-section (PI_CS0_ and PI_CSm,_ respectively); minimal and maximal densities of active reaction centers per leaf cross-section (RC/CS_0_ and RC/CS_m_, respectively); the quantum yield of electron transport (φEo); and the efficiency of the electron transfer from QA- to QB (ψo)

	REC	EL	F_**v**_**/F**_**m**_	ψo	φEo	PI	PI_**CS0**_	PI_**CSm**_	ABS/CS	TR_**0**_**/CS**	ET_**0**_**/CS**	DI_**0**_**/CS**	ABC/RC	TR_**0**_**/RC**	ET_**0**_**/RC**	DI_**0**_**/RC**	RC/CS_**0**_	RC/CS_**m**_
REC		-	0.52	0.47	0.56	0.45	0.51	0.52	0.34	0.58	0.62	− 0.35	− 0.32	-	0.57	− 0.32	0.54	0.61
EL	-		− 0.25	− 0.28	− 0.21	-	-	-	0.36	-	-	0.48	-	0.45	-	0.40	-	-

EL was significantly negatively correlated with the individual chlorophyll *a* fluorescence parameters: *F*_v_/*F*_m_, *Ψ*_0_, and φE_0_. Positive correlation was statistically significant from 0.36 to 0.48 between EL and the parameters: ABS/CS, DI_0_/CS, TR_0_/RC, and DI_0_/RC (Tqb. 3).

### Quantitative trait loci (QTL) for plants recovery

**Fig. 3:**
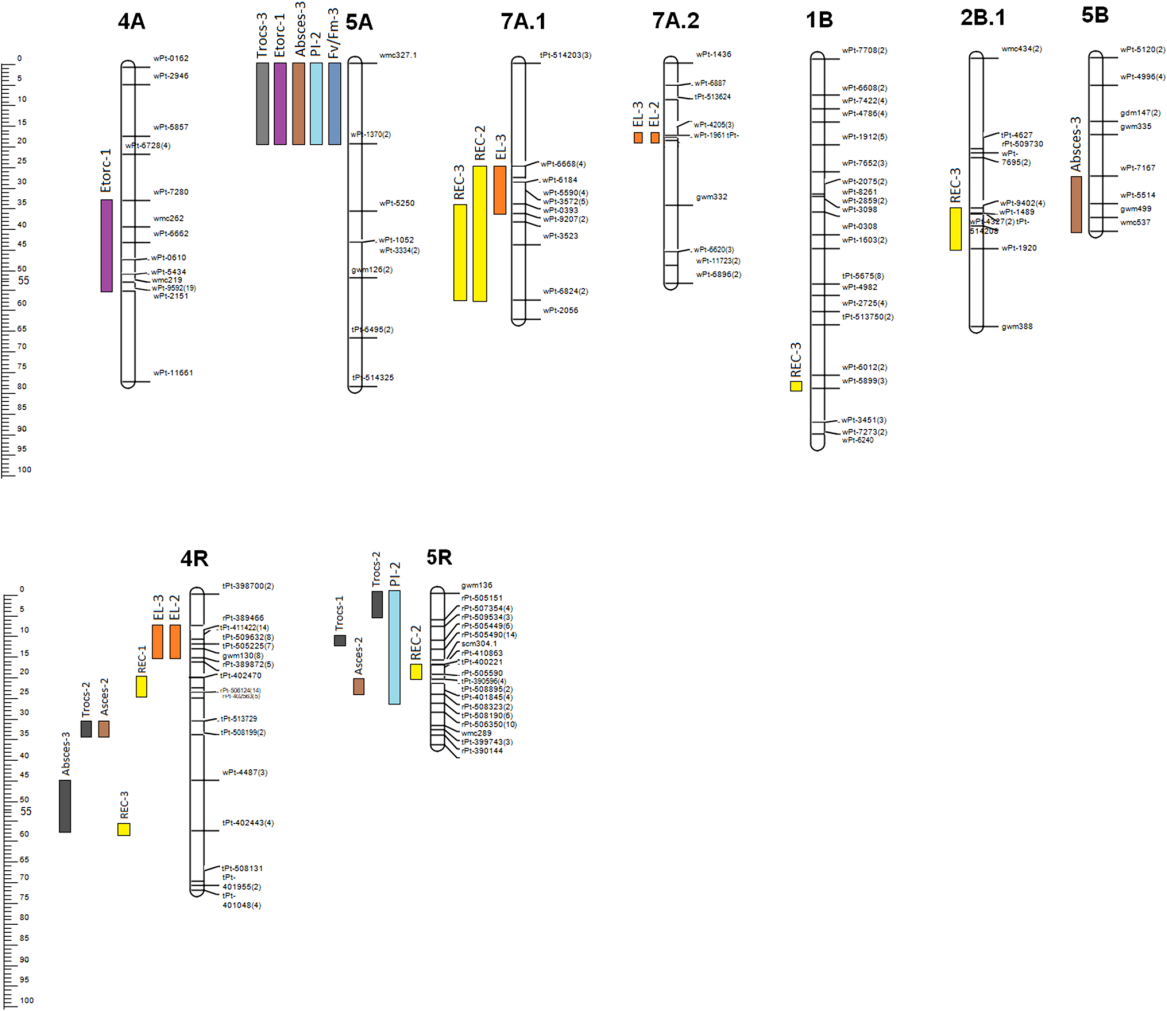
Interval map (cM) for 4A, 5A, 7A.1, 7A.2, 1B, 2B.1, 5B, 4R, and 5R chromosomes of ‘Hewo’ x ‘Magnat’ DH population with QTL identified by CIM method for plant recovery (REC), and electrolyte leakage (EL), Fv/Fm, PI and energy fluxes parameters

Using CIM analysis, QTL regions for recovery (REC) expressed as percentage of survived plants were identified on chromosomes 7A.1, 1B, 2B.1, 4R, and 5R (Table 4; Fig. 3). Additionally, all QTL regions identified by SMA method are shown in Table S3. The phenotypic variation explained by those QTL ranged from 4.4 to 15.8% depending on winter conditions and method used (Table 4; Table S3). The QTL for percentage of survived plants evaluated in two experiments were co-located on chromosome 7A.1 and 1B and named *Qfr.hm-7A.1* and *Qrec.hm-1B.1*, respectively (Table 4; Fig. 3). Those loci covered 27.5–57.2 cM distance for *Qfr.hm-7A.1* and explained up to 9.5% of phenotypic variation. For second locus, *Qrec.hm-1B.1* CIM analysis showed only one marker *wPt-5899(3)* in experiment 3 (Table 4; Fig. 3). Those loci explained up to 8.2% of phenotypic variation with the LOD value 2.5 (Table 4).

**Table 4:** Summary of QTL identified using CIM method for plant recovery (REC), percentage of survived plants and electrolyte leakage (EL) from leaves after plant freezing

QTL name	QTL name	Trait-experiment	Flanking markers	Interval/position (cM)	LOD	*R*^2^ (%)^a^	Add^b^	Favorable allele^c^
Qel.hm-7A-1.4	*Qfr.hm-7A.1*	EL-3	*wPt-6668(4)*-*wPt9207(2)*	27.5–38.3	3.1	9.5	− 5.5	M
Qrec.hm-7A-1.4		REC-2	*wPt-6668(4)*-*wPt-6824(2)*	27.5–57.2	2.6	6.9	6.6	H
		REC-3	*wPt-0393*-*wPt-6824(2)*	36.2–57.2	2.5	9.2	6.5	H
Qel.hm-7A-2.1	*Qel.hm-7A.2*	EL-2	*tPt-513624*-*tPt-2230(2)*	8.9–19.9	2.6	8.7	8.1	H
		EL-3	*tPt-513624*-*wPt-1961*	8.9–19.3	4.1	13.0	6.4	H
Qrec.hm-1B.1	*Qrec.hm.1B.1*	REC-3	*wPt-5899(3)*	79.9	2.5	8.2	6.7	H
Qrec.hm-2B.1	*Qrec.hm-2B.1*	REC-3	*wPt-9402(4)*-*wPt-1920*	36.3–46.1	2.9	10.4	7.2	H
Qel.hm-4R.1	*Qel.hm-4R.1*	EL-2	*rPt-389466*-*rPt-389872(5)*	9.8–16.5	6.5	22.8	18.7	H
		EL-3	*rPt-389466*-*rPt-389872(5)*	9.8–16.5	2.5	7.7	4.9	H
Qrec.hm-4R.1	*Qrec.hm-4R.2*	REC-1	*tPt-402470*-*rPt402563(5)*	22.6–25.2	3.5	12.9	-0.8	M
Qrec.hm-4R.2	*Qrec.hm-4R.3*	REC-3	*tPt-402443(4)*	57.2	3.0	10.8	− 10.8	M
Qrec.hm-5R.1	*Qrec.hm-5R.1*	REC-2	*rPt-508323(2)*-*tPt-390596(4)*	22.2–26.5	2.5	6.3	− 6.9	M
Qel.hm-5R.1	*Qel.hm-5R.2*	EL-3	*wmc289*-*rPt-506350(10)*	32.0–32.9	2.5	5.0	6.8	H

^a^*R*^2^ (%), the percentage of phenotypic variance explained by the QTL

^b^Add, additive effects of QTL expressed in the trait unit

^c^Favorable allele for each QTL: H, cv. Hewo and M, cv. Magnat

### Quantitative trait loci (QTL) for electrolyte leakage

Electrolyte leakage (EL) was measured two times during winter 2012/2013. QTL controlling EL after freezing, identified by CIM method were on chromosomes 7A.1, 7A.2, 4R, and 5R (Table 4; Fig. 3). Additionally, all QTL regions identified by SMA method are shown in Table S4. The phenotypic variation explained by those QTL ranged from 3.3 to 22.8% depending on experiment and method used (Table 4; Table S4). The locus *Qfr.hm-7A.1*, which was identified as associated with EL in experiment 3 was also co-located with QTL identified for plants recovery on chromosome 7A.1. Locus *Qfr.hm-7A.1* explained 9.5% of phenotypic variation observed for EL (Table 4) with LOD value 3.1 and was in similar position in cM to loci identified for plants recovery (Table 4). Another locus identified on chromosome 7A, *Qel.hm-7A.2* was located in 8.9–19.9 cM distance and explained up to 13% of phenotypic variation; it contained loci found in experiment 2 and 3 (Table 4; Fig. 3). Locus *Qel.hm-4R.1* located in 9.8–16.5 cM distance on chromosome 4R was also identified in experiment 2 and 3 (Table 4; Fig. 3). It explained up to 22.8% of phenotypic variation with the LOD value 6.5 in experiment 2 (Table 4). One loci identified in experiment 3 was located on chromosome 5R and covered by *wmc289* to *rPt-506350(10)* markers (Table 4; Fig. 3). This loci explained 6.8% of phenotypic variation (Table 4).

### Quantitative trait loci (QTL) for chlorophyll a fluorescence parameters

QTL for chlorophyll *a* fluorescence parameters identified by CIM method were located on chromosomes 4A, 5A, 5B, 4R, and 5R (Table 5; Fig. 3). Additionally, QTL identified by SMA method are shown in Tables S5–S7.

**Table 5:** Summary of QTL for Fv/Fm (the yield of the energy trapping in PSII), PI (performance indexes of PSII) and energy fluxes (RC per single, active PSII reaction center and CS per leaf cross-section) identified using CIM methods. QTL name includes each parameter name (after the letter Q)

QTL	QTL name	Trait-experiment	Flanking markers	Interval/position (cM)	LOD	*R*^2^ (%)^a^	Add^b^	Favorable allele^c^
Qetorc.hm-4A.1	*Qchl.hm-4A.1*	ET_0_/RC-1	*wPt-7280*-*wPt-2151*	32.3–56.6	2.5	9.1	3.7	H
Qfvfm.hm-5A.2	*Qchl.hm-5A.1*	Fv/Fm-3	*wmc327.1*-*wPt-1370(2)*	0.0–19.4	3.3	12.0	0.1	H
Qpi.hm-5A.2		PI-2	*wmc327.1*-*wPt-1370(2)*	0.0–19.4	3.8	11.5	0.1	H
Qabscs.hm-5A.2		ABS/CS-3	*wmc327.1*-*wPt-1370(2)*	0.0–19.4	3.3	12.7	25.5	H
Qetocs.hm-5A.2		ET_0_/CS-3	*wmc327.1*-*wPt-1370(2)*	0.0–19.4	3.9	13.0	11.4	H
Qtrocs.hm-5A.2		TR_0_/CS-3	*wmc327.1*-*wPt-1370(2)*	0.0–19.4	5.4	19.6	36.8	H
Qabscs.hm-5B.1	*Qchl.hm-5B.1*	ABS/CS-3	*wPt-7167*-*wmc537*	28.6–41.9	3.9	11.6	24.8	H
Qabscs.hm-4R.2	*Qchl.hm-4R.1*	ABS/CS-2	*tPt-513729*-*tPt-508199(2)*	32.9–33.9	2.5	7.5	− 11.3	M
Qtrocs.hm-4R.2		TR_0_/CS-2	*tPt-513729*-*tPt-508199(2)*	32.9–33.9	2.8	8.8	− 9.0	M
Qabscs.hm-4R.2		ABS/CS-3	*wPt-4487(3)*-*tPt-402443(4)*	45.0–57.2	3.1	10.3	− 22.8	M
Qpi.hm-5R.1	*Qchl.hm-5R.1*	PI-2	*gwm136-tPt*-*508190(6)*	0.0–28.7	3.2	9.6	0.1	H
Qtrocs.hm-5R.1		TR_0_/CS-2	*gwm136-rPt*-*507354(4)*	0.0–8.0	3.1	10.8	− 10.6	M
Qabscs.hm-5R.1		ABS/CS-2	*tPt-390596(4)*-*rPt-508323(2)*	22.2–26.5	2.5	7.5	− 11.8	M
Qtrocs.hm-5R.1		TR_0_/CS-1	*rPt-505449(6)*	13.5	2.6	6.2	− 6.1	M

^a^*R*^2^ (%), the percentage of phenotypic variance explained by the QTL

^a^Add, additive effects of QTL expressed in the trait unit

^c^Favorable allele for each QTL: H, cv. Hewo and M, cv. Magnat

Abbreviations: *ABS/CS*, absorbed energy flux per leaf cross-section (CS); *Tr*_*0*_*/CS*, trapped energy flux in PSII reaction centers per leaf cross-section; *ET*_*0*_*/CS*, the energy flux for electron transport per leaf cross-section; *ET*_*0*_*/RC*, the energy flux for electron transport per the single, active PSII reaction center; *F*_*v*_*/F*_*m*_, yield of the energy trapping in PSII; *PI*, performance indexes of PSII

Locus *Qchl.hm-4A.1* identified on chromosome 4A in a distance between 21.1 cM to 56.6 cM consisted QTL for ET_0_/RC and explained 9.1%of phenotypic variation with the LOD value 2.5 (Table 5; Fig. 3). Locus *Qchl.hm-5A.1* covered by *wmc327.1* to *wPt-1370(2)* markers was identified for *Fv/Fm*, PI, ABS/CS, ET_0_/CS and TR_0_/CS in experiment 2 and 3 (Table 5; Fig. 3). It explained up to 19.6% of phenotypic variation with the LOD value up to 5.4 for TR_0_/CS (Table 5). On chromosome 5B, locus *Qchl.hm-5B.1* was identified for ABS/CS with the LOD value 3.9 and phenotypic variation 11.6% (Table 5; Fig. 3). Locus *Qchl.hm-4R.1* was identified on chromosome 4R for TR_0_/CS measured in experiment 2 and ABS/CS for experiment 2 and 3 (Table 5; Fig. 3). Loci identified for ABS/CS and TR_0_/CS measured in experiment 2 were located in the same position (32.9–33.9 cM; Table 5). On chromosome 5R, locus *Qchl.hm-5R.1* was identified for PI, TR_0_/CS and ABS/CS measured in experiment 2 and TR_0_/CS measured in experiment 1 (Table 5; Fig. 3). This QTL explained up to 10.8% of phenotypic variation with LOD value up to 3.2 (Table 5).

### Candidate genes for analyzed traits

Seven candidate genes were in silico identified within QTL found for plants recovery (REC) and electrolyte leakage (EL) as well as trapped energy flux in PSII reaction centers per leaf cross-section (Tr_0_/CS), the energy flux for electron transport per leaf cross-section (ET_0_/CS) and absorbed energy flux per leaf cross-section (ABS/CS) traits (Table 6). Gene coding PPR protein involved in chloroplast RNA processing, modification, and splicing was identified as associated with plants recovery (Table 6). Genes identified as associated with electrolyte leakage encode mRNA-binding protein BTR1-like and involved in regulation of gene expression as well as transmembrane cyclic nucleotide-gated ion channel (Table 6). In turn, genes associated with chlorophyll *a* fluorescence parameters were chloroplastic uridine kinase-like protein 1 involved in the pyrimidine salvage pathway, uncharacterized LOC119301557 and phosphoinositide phosphatase SAC9 involved in stress signaling (Table 6).

**Table 6:** The characteristics of candidate genes identified in the QTL regions associated with the seedlings traits: electrolytes leakage (EL), plants recovery (REC), trapped energy flux in PSII reaction centers per leaf cross-section (Tr0/CS), the energy flux for electron transport per leaf cross-section (ET0/CS) and absorbed energy flux per leaf cross-section (ABS/CS) in DH ‘Hewo x Magnat’ lines mapping population studied

QTL name	Trait/experiment	Flanking marker	Sequence query	Position	Sequence ID (mRNA)	Reference organism	Predicted protein	Predicted function
Qel.hm-7A.2	EL-2 EL-3	*tPt-513624*	TraesCS7A03G0506500LC (low confidence)	Chr7A:189,793,060..189793458 (+ strand)	XM_037604689.1	*Triticum dicoccoides*	Uncharacterized LOC119331524 transcript var. X2	BTR1-like protein, mRNA binding, regulation of gene expression
Qel.hm-5R.2	EL-3	*rPt-506350*	SECCE5Rv1G0349830 (high confidence)	Chr5R:686,819,682..686824096 (− strand)	XM_037587241.1	*Triticum dicoccoides*	Nucleotide-gated ion channel 14 (LOC119311587)	Voltage-gated potassium channel activity, cyclic nucleotide-gated ion channel, integral component of membrane, transmembrane helical protein
Qfr.hm-7A.1	REC-3	*wPt-0393*	TraesCS4B01G034600 (high confidence)	Chr4B:25,384,962..25394077 (− strand)	XM_037576333.1	*Triticum dicoccoides*	Pentatricopeptide repeat-containing protein At4g18520, chloroplastic-like (LOC119299015), transcript var. X1	PPR protein, involved in chloroplast RNA processing, modification and splicing
Qchl.hm-4A.1	ET_0_/RC-1	*wPt-2151*	TraesCS4A03G1247800 (high confidence)	Chr4A:752,456,325..752459406 (− strand)	XM_020328553.1	*Aegilopstauschii* subsp. *strangulata*	Uncharacterized LOC109769846	-
Qchl.hm-5B.1	ABS/CS-3	*wPt-7167*	TraesCS5B03G0735400 (high confidence)	Chr5B:473,178,495..473184426 (− strand)	XM_037586621.1	*Triticum dicoccoides*	Uridine kinase-like protein 1, chloroplastic (LOC119310993)	Involved in the pyrimidine salvage pathway
Qchl.hm-5R.1	TR_0_/CS-2	*rPt-507354*	SECCE5Rv1G0327250 (high confidence)	Chr5R:490,868,494..490871516 (− strand)	XM_037578543.1	*Triticum dicoccoides*	Uncharacterized LOC119301557	Gag-Pol polyprotein/retrotransposon, transmembrane helix protein
	TR_0_/CS-1	*rPt-505449*	SECCE5Rv1G0298360 (high confidence)	Chr5R:11,157,843..11168368 (+ strand)	XM_037576070.1	*Triticum dicoccoides*	Phosphoinositide phosphatase SAC9 (LOC119298775), transcript var. X3	Probable phosphoinositide phosphatase that could be involved in stress signaling, phosphoric ester hydrolase activity, phosphatidylinositol metabolic process, response to osmotic stress, integral component of membrane

## Discussion

Recently, climate change has been disturbing the natural hardening process (Dalmannsdottir et al. 2017). Global warming caused by the increase in average temperature on Earth is reflected in climate instability and violent local breakdowns of the weather. This leads to the limitation of seamless adjustment of plant physiology to the prevailing conditions, including the course of winters (Rapacz et al. 2014; Dalmannsdottir et al. 2017). Thus, an effective method to evaluate plant freezing tolerance and/or winter hardiness under unstable winter conditions is urgently needed. In our study, we observed differences in plant recovery of DH ‘Hewo x Magnat’ lines population after freezing depending on the experiment condition. Furthermore we observed that differences in plant recovery between those DH lines were closely related to damages of photosynthetic apparatus measured by means of JIP analysis. The relationship between plant survival and photochemical efficiency of PSII under unstable winter conditions was confirmed earlier in wheat (Clement and van Hasselt 1996; Rapacz 2007), oat (Rizza et al. 2001), barley (Francia et al. 2004), and triticale (Rapacz et al. 2011, 2015ab).

The most commonly reported fluorescence parameter for evaluating plant responses to abiotic stress is *F*_*v*_*/F*_*m*_ which informs about the maximum photochemical PSII activity (Del Rosso et al. 2009). In the studied population, a high positive and statistically significant correlation (0.52) between plant survival and *F*_*v*_*/F*_*m*_ was obtained. However, there are many reports available showing that different parameters of chlorophyll fluorescence parameters followed by JIP tests could be used as the more effective indicators of freezing tolerance than *F*_*v*_*/F*_*m*_, especially when plants are well cold acclimated before the measurements, which was also reported for triticale (Rapacz et al. 2011, 2015ab, 2017). In the present study, we confirmed high statistically significant correlation values of the remaining JIP parameters with plant survival. Similarly to Rapacz et al. (2015a) study, in our results on the JIP test, best correlating with plant survival were ET_0_/CS and RC/CS_m_ parameters. Moreover, in our study, the dependence of plants ability to recover after freezing on photosynthetic apparatus was also reflected in the colocation of quantitative traits loci on chromosomes 4R and 5R, where many genes related with freezing tolerance were earlier identified.

The location of QTL that control the photochemical efficiency of PSII on chromosome 5 group (5A, 5B, and 5R) may indicate that this trait is partially controlled by the frost-resistance genes. The main QTL associated with photochemical activity of PSII were mapped on chromosome 5A at 58.7–78.1 cM, within the range for which the major frost-resistance loci *Fr-A1* and *Fr-A2* identified by other authors (Vágújfalvi et al. 2000, 2003). The QTL identified on chromosome 5A for chlorophyll fluorescence parameters may also contain the *vrn1* gene (located in close proximity to the *Fr-A1* frost-resistance locus). This hypothesis may be supported by the fact that the *wPt-1370* marker located on chromosome 5A in DH Hewo x Magnat mapping population used in our study was reported as a marker in close proximity to the gene *vrn1* on the genetic map developed for the wheat population Berkut/Krichauff (Genc et al. 2010). On chromosome 5A, the following genes have been also identified: *Cor14b*, *Cbf3* (Vágújfalvi et al. 2000, 2003; Miller et al. 2006), *Cbf14,* and *Cbf15* (Båga et al. 2007). In addition, the *Cbf* genes: *TmCBF12* and *HvCbf14* recognized as candidate genes for frost-resistance genes were also located on chromosome 5A at the locus labeled *Fr-A2* in wheat and respectively at the locus labeled *Fr-H2* in barley at the homeological chromosome 2H (Knox et al. 2008; Fricano et al. 2009). On the 5B wheat chromosome, genes associated with flowering and frost tolerance have been identified and the locus was labeled *Fr-B1* (Toth et al. 2003). These results clearly indicate an important role of 5 chromosome groups in the creating of plant freezing tolerance not only in wheat but also in triticale (Alm et al. 2011).

For the purpose of present QTL analysis, new genetic map of DH ‘Hewo’ x ‘Magnat’ lines population was developed. The unique set of SSR and DArT markers enabled to assign a total of 721 markers into 22 linkage groups of triticale. Diversity Arrays Technology (DArT) used in our research is known as a tool used to construct genetic maps of many crop species (Wenzl et al. 2004). That microarray technology can provide the information of several thousand sequence-specific markers without sequence information (Jaccoud et al. 2001; Karbarz et al. 2020). In our research, the distribution of DArT and SSR markers showed the highest saturation of R genome in contrast to the A and B genomes. Similar effect with the best coverage and highest density of R genome in triticale genetic maps were previously described by Tyrka et al. (2011) and Karbarz et al. (2020). Up to now, several genetic maps have been developed and reported QTL analysis in triticale (Alheit et al. 2011; Badea et al. 2011; González et al. 2005; Tyrka et al. 2011, 2015; Krzewska et al. 2012, 2015; Dyda et al. 2019; Karbarz et al. 2020).

Numerous QTL associated with the most commonly analyzed *F*_*v*_*/F*_*m*_ chlorophyll *a* fluorescence parameter have been identified in association with various types of stress (Yang et al 2007; Liang et al 2010; Alm et al. 2011; Zhang et al. 2017; Czyczyło-Mysza et al. 2011, 2013; Kumar et al. 2012; Cheng et al. 2012; Bhusal et al. 2018; Dyda et al. 2019). QTL for *F*_*v*_*/F*_*m*_ under drought stress was identified in wheat on chromosome 5A (Czyczyło-Mysza et al. 2011; Liang et al. 2010; Alm et al. 2011). No correlation was observed in our study between plant recovery after freezing and electrolyte leakage from freezing-damaged leaf tissues of triticale. On the other hand, 10 QTL regions related to the stability of cell membranes measured by electrolyte leakage from leaf tissues after freezing have been identified. Further studies would help assess whether the identified QTLs for the EL could be related to other parameter/s related to plant survival after freezing that was not measured in this work. The QTL identified for freezing tolerance of photosynthetic apparatus was located on chromosomes inherited from wheat, 4A, 5A, 5B, and on rye chromosomes, 4R and 5R. QTL on chromosomes 4A, 5A, and 5B, two loci on chromosome 4R as well as loci on 5R chromosome were identified in both experiments conducted during winter 2012/2013. In our study, loci related to cell membrane stability were identified on chromosome 7A, that is the same chromosome where Morgan and Tan (1996) identified QTL associated with osmoregulation in wheat. In rice (*Oryza sativa* L.) under drought stress, QTL of the cell membranes stability were identified on chromosomes 1, 3, 7, 9, 11, and 12 (Tripathy et al. 2000) as well as chromosome 8 (Lilley et al. 1996; Tripathy et al. 2000). The literature also provides a lot of information on the genes that are expressed in response to the stress of drought and low temperature and regulate the condition of cell membranes (Danyluk et al. 1998; Nylander et al. 2001; Alm et al. 2011; Kocheva et al. 2014; Janeczko et al. 2019).

In our research, we mainly focused on finding the molecular background of freezing tolerance based on the crosstalk between chlorophyll fluorescence measurements of photosynthetic apparatus freezing tolerance, cell membrane stability, and plant recovery abilities after freezing. For these three components of plant freezing tolerance, we found durable, strong, constant quantitative trait loci regardless on plant growth conditions. A total of 9 genomic regions (QTL on 7A, 1B, 2B, 4R and 5R chromosomes) associated with plant survival after freezing were identified. Four of them were mapped in at least two experiments (7A, 1B and one locus on 4R chromosome). In contrast, QTL identified for one seasons can be considered as potential loci and/or loci specific to particular weather conditions. QTL of plant survival after freezing identified on chromosome 1B confirmed previous results obtained for Norstar × Cappelle-Desprez DH population where locus of frost resistance was mapped (Chodaparambil 2009). Frost-resistance loci in the DH Norstar × Cappelle-Desprez population were identified on chromosome 1B at 90–118 cM. In the present study, QTL of plant recovery was located in a close position on chromosome 1B at position 79.9 cM. Similar to the results obtained for the Hewo × Magnat DH population, also some of the earliest studies have indicated that genes associated with wheat frost resistance are located on chromosomes 7A and 2B (Sutka 1981; 1994; Galiba et al. 2003) and additionally on chromosomes: 4B and 4D (Law and Jenkins 1970; Puchkov and Zhirov 1978; Sutka 1981; Roberts 1986). Chromosome groups 1, 2, and 7 indicated in this study as containing strong QTL of plant survival regardless of environmental conditions, could mainly carry loci responsible for vernalization, flowering and photoperiod regulation, which only may indirectly affect cereal frost resistance (Galiba et al. 1995; Law and Worland 1997; Mahfoozi et al. 2000; Skinner et al. 2004). Genes related to the vernalization process are located, for example, on the wheat group 1 homeological chromosome (*vrn3* gene) (Law and Worland 1997) and on barley 7 chromosomes (*sh2* gene) (Pan et al. 1994; Galiba et al. 1995) and on the 7 rye chromosome (Plaschke et al. 1993; Galiba et al. 1995). In the presented study, QTL of plant survival after freezing was located exclusively on one of the chromosomes of 5 group, rye chromosome (5R). The reason why the locus responsible for plant survival was not mapped on chromosome 5A in the studied population is probably the lack of variation between parents in this region. Moreover, in triticale the absence of important freezing tolerance loci on wheat chromosome 5D are reported to be compensated by 5R loci.

On chromosome 5R many authors have identified genes of freezing tolerance described as CBF family, closely related to the locus corresponding to the locus *Fr2* on homeological chromosomes of the 5 group in barley, diploid, and hexaploid wheat and meadow fescue (Francia et al. 2007; Baga et al. 2007; Knox et al. 2008; Tamura and Yonemaru 2010; Alm et al. 2011; Zhang et al. 2019). Campoli et al. (2009) also identified 12 different *Cbf* family genes on the long arm of the 5R chromosome. Loci *ScCbf* (*ScCbf2*, *ScCbf6*, *ScCbf9b*, *ScCbf12*, *ScCbf15*, *ScIce2*, *ScDhn3*) were mapped on chromosome 5R in similar positions to the frost-resistance loci *Fr-H2*/*Fr-am2* in barley and diploid wheat, respectively (Li et al. 2011a, b). The described *Cbf* family genes (Campoli et al. 2009; Li et al. 2011a, b) were located on the 5R chromosome in similar position as *Qrec.hm-5R.1* identified in our study. Campoli et al. (2009) clearly showed that the expression of *Cbf* family genes is closely related to the temperature during the acclimation process and the measurement time. This hypothesis could explain why this QTL was identified in our study only in experiment 3.

In the evaluated Hewo × Magnat DH population, QTL regions co-located for plant survival, membrane stability, and photochemical PSII efficiency were identified on rye chromosomes 4R and 5R. QTL for these parameters could indicate the partially general genetic background responsible for the survival of whole plants and the protection of their photosynthetic apparatus after freezing. The colocation of the QTL for photochemical PSII efficiency, membrane stability and plant survival may be due to the fact that co-located genes can control the effectiveness of photosynthesis under stress conditions and affect protection during acclimatization or to aid plant regeneration after stress. The involvement of such mechanisms can be jointly controlled at the genome level, and even the efficiency of the photosynthetic apparatus can directly be responsible for the ability of plant to survive freezing. On the other hand, the incomplete correlation between the chlorophyll fluorescence parameters, membrane stability and the plant survival and the lack of collocation of many QTL obtained between these parameters suggest that not all physiological mechanisms were indicated and plant recovery, membrane stability, and photosynthetic efficiency of PSII under stress conditions in many aspects are controlled by different genes.

Our results of QTL and gene identification strongly indicate physiological and genetic relationship of the plant survival after freezing with the photochemical activity of the photosystem II. For plants recovery after freezing stress, gene coding pentatricopeptide repeat-containing protein At4g18520, involved in chloroplast RNA processing, modification, and splicing, was identified. Such a result indicates a significant role of chloroplast genes in winter triticale seedlings frost survival. Other authors research, conducted in *Arabidopsis thaliana* showed that protein At4g18520 may be required for proper chloroplast development, the regulation of the plastid gene expression probably through regulation of plastid-encoded polymerase (PEP) dependent chloroplast transcription, for RNA editing of several chloroplastic transcripts (especially accD transcripts), for processing of the chloroplastic rpoA pre-mRNA as well as for the monocistronic rpoA transcript processing (Yin et al. 2012).

In turn, genes identified as associated with chlorophyll *a* fluorescence parameters were chloroplastic uridine kinase-like protein 1, uncharacterized LOC119301557, and phosphoinositide phosphatase SAC9. Uridine kinase is plasma membrane protein, involved in CTP and UMP salvage, nucleoside metabolic process, protein secretion, regulation of exocytosis, and vesicle docking involved in exocytosis. Phosphoinositide phosphatase could be involved in stress signaling (Williams et al. 2005). At the same time, these genes were identified for the chlorophyll fluorescence parameters, the values of which showed a positive correlation with the plants recovery tests result. Genes found for electrolyte leakage code mRNA-binding protein BTR1-like involved in regulation of gene expression as well as transmembrane cyclic nucleotide-gated voltage-gated potassium channel. Protein-forming potassium channel was identified among abiotic stress responsive proteins of wheat grain determined using proteomics technique by Kamal et al. (2010).

In conclusion, QTL that control the PSII photochemical efficiency were identified on chromosome 5 group (5A, 5B, and 5R). Six genomic regions associated with plant survival after freezing (REC) were identified and two of them were mapped in two experiments (chromosomes 7A.1, 1B, 5R, and 4R). Co-located QTL for plant survival, membrane stability, and photochemical PSII efficiency were identified on chromosomes 4R and 5R. The collinearity of the QTL for PSII photochemical efficiency, membrane stability, and plant survival may be due to the fact that co-located genes can control the effectiveness of photosynthesis under stress conditions and affect protection during acclimatization or to aid plant regeneration after stress. The same QTL for electrolyte leakage and plant recovery *Qfr.hm-7A.1* was identified in two experiments indicating that these QTL are responsible for differences in freezing tolerance in our mapping population regardless on strong genotype-environmental interaction observed for freezing tolerance in triticale making this regions particularly interesting for breeders. QTL for percentage of plant survival and electrolyte leakage were both co-located on chromosomes 4R and 5R with QTL for several chlorophyll fluorescence parameters. Such results indicate that the correlation exist between freezing tolerance of photosynthetic apparatus, plasma membranes and plant regrowth after freezing. This conclusion is also supported by chloroplast and membrane genes identified in genome regions associated with these traits.

## Supplementary Information

Below is the link to the electronic supplementary material.

Figure S1 A. Genetic linkage map of A chromosomes with molecular markers. (JPG 3498 KB)

Figure S1 B. Genetic linkage map of B chromosomes with molecular markers. (JPG 5346 KB)

Figure S1 R. Genetic linkage map of R chromosomes with molecular markers. (JPG 5349 KB)

Figure S2. Mean values of chlorophyll a fluorescence parameters measured in DH population after cold acclimation in three different field conditions. A, B, C, D, E, F, G, H, I, J charts were made for ABS/RC, TR0/RC, ET0/RC, DI0/RC, ABS/CS, TR0/CS, ET0/CS, DI0/CS, FV/FM and PI, respectively. Plant survival on the x-axis (0-100) is a number of surviving (regrowing) plants examined after three weeks of regrowth in glasshouse (temp. about +15°C) and expressed as a percentage of plant survival. (DOCX 1369 KB)

Table S1. Genetic map and segregations of markers in population ‘Hewo’ x ‘Magnat’. Number of cosegregating markers was given (in brackets) in the names of representing bin markers. Segregartions of redundant markers were not provided. Physical positions of merkers were obtained afret balstn of marker sequences on ChineseSpring wheat V1.0 (URGI: https://wheat-urgi.versailles.inra.fr/Seq-Repository/BLAST ) and Lo1 rye chromosomes (WheatOmics 1.0: http://202.194.139.32/blast/blast.html). (XLSX 244 KB)

Table S2. Data on maximum expected number of recombinations per individual (Max_rec) extracted and sum of adjacent recombination frequencies (Mean_rec) from JoinMap. Percentage of lines without crossing-over (c-o) was calculated in Excel. (DOCX 19 KB)

Table S3. Summary of QTL identified using SMA method for plants recovery REC (percentage of survived plants after freezing) after freezing test performed during winter 2011/2012 (Experiment 1) and 2012/2013 (Experiments 2, 3).Table S4. Summary of QTL for electrolyte leakage (EL) from leaves after freezing identified using SMA method. Table S5. Summary of QTL for Fv/Fm (the yield of the energy trapping in PSII) and PI (performance indexes of PSII) identified using SMA method. QTL name includes each parameter name (after the letter Q). Table S6. Summary of QTL for energy fluxes (per single, active PSII reaction center; RC) identified using SMA method. QTL name includes each parameter name (after the letter Q). Abbreviations: ABS/RC - absorbed energy flux per the single, active PSII reaction center (RC); TR0/RC - trapped energy flux in PSII reaction centers per the single, active PSII reaction center; DI0/RC - dissipation of energy in PSII reaction centers per the single, active PSII reaction center. Table S7. Summary of QTL for phenomenological energy fluxes (per leaf cross-section; CS) identified using SMA method. QTL name includes each parameter name (after the letter Q). Abbreviations: ABS/CS - absorbed energy flux per leaf cross-section (CS); Tr0/CS - trapped energy flux in PSII reaction centers per leaf cross-section; ET0/CS - the energy flux for electron transport per leaf cross-section; DI0/CS – dissipation of energy in PSII reaction centers per leaf cross-section. (DOC 227 KB)

## Data Availability

Not applicable.
