# Supplementary figures and images for: Quantitative trait loci and candidate genes associated with freezing tolerance of winter triticale (× Triticosecale Wittmack)
Source: J Appl Genet. 2021 Sep 7;63(1):15–33. doi: 10.1007/s13353-021-00660-1 (PMC8755666; doi:10.1007/s13353-021-00660-1)

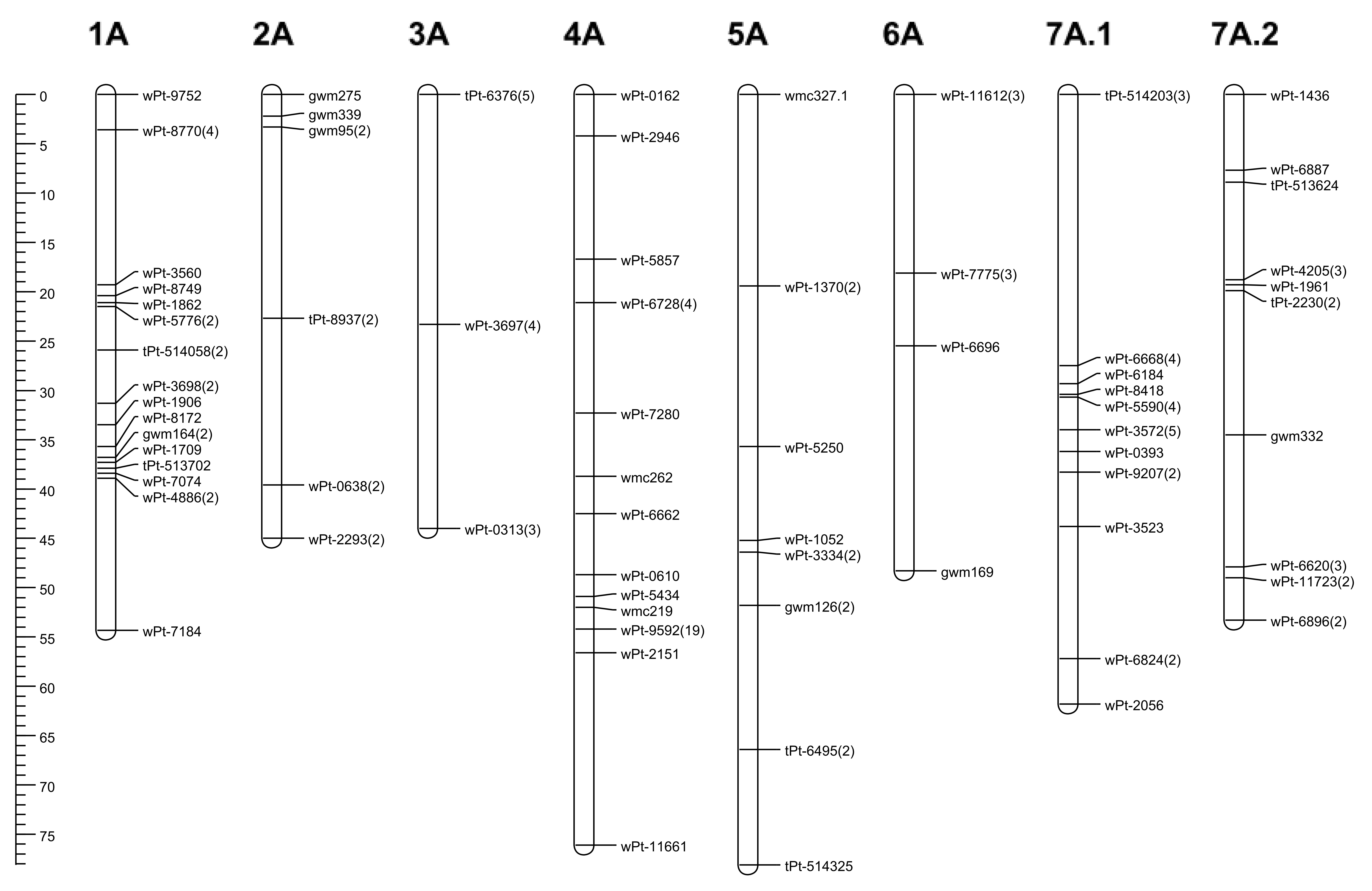

Supplement: Supplementary file 1 — Genetic linkage map of A chromosomes with molecular markers. (JPG 3498 KB) [file 13353_2021_660_MOESM1_ESM.jpg]

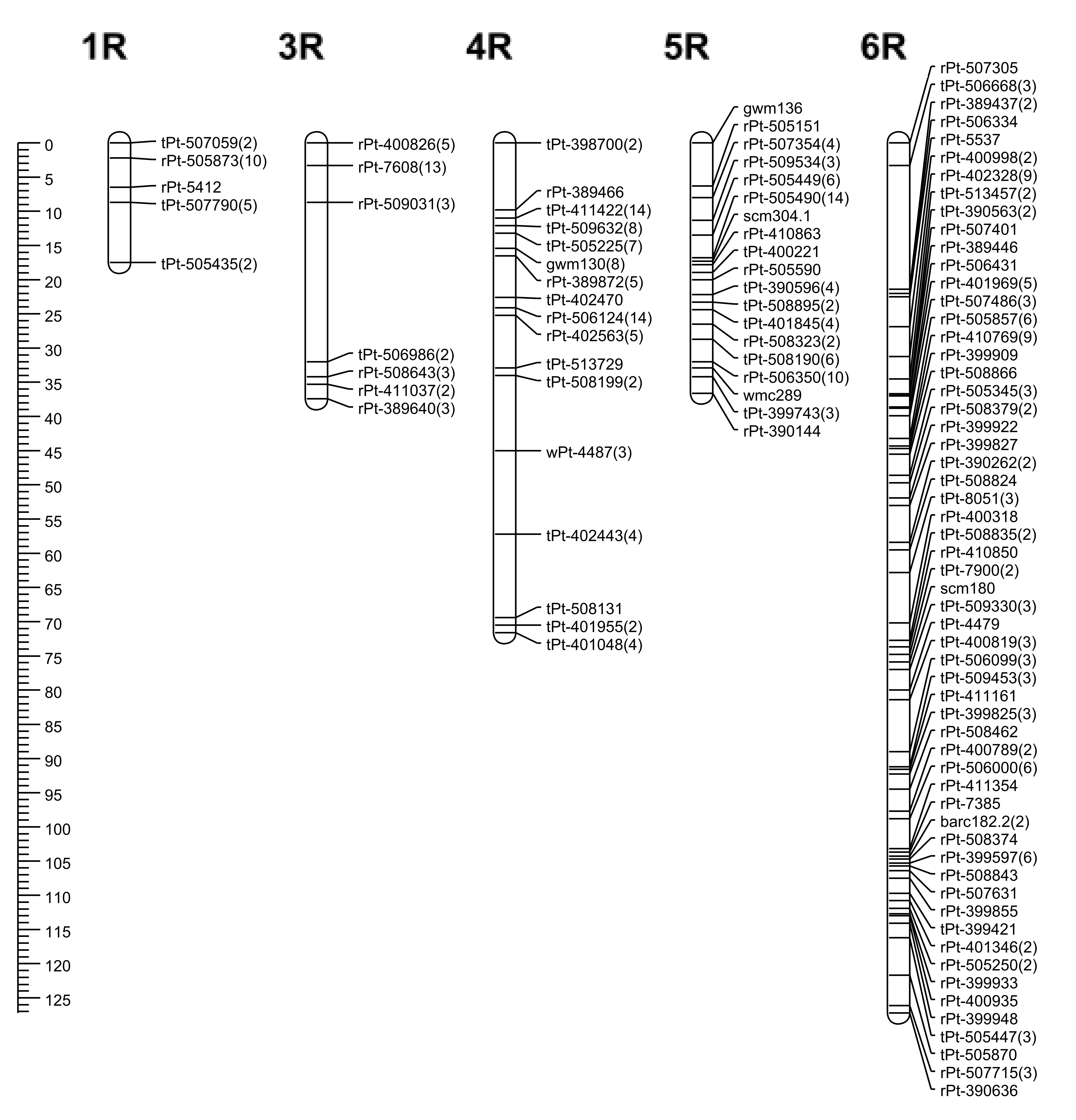

Supplement: Supplementary file 3 — Genetic linkage map of R chromosomes with molecular markers. (JPG 5349 KB) [file 13353_2021_660_MOESM3_ESM.jpg]
