# Supplementary material for: Quantitative trait loci and candidate genes associated with freezing tolerance of winter triticale (× Triticosecale Wittmack)
Source: J Appl Genet. 2021 Sep 7;63(1):15–33. doi: 10.1007/s13353-021-00660-1 (PMC8755666; doi:10.1007/s13353-021-00660-1)
Supplement: Supplementary file 4 — Mean values of chlorophyll a fluorescence parameters measured in DH population after cold acclimation in three different field conditions. A, B, C, D, E, F, G, H, I, J charts were made for ABS/RC, TR0/RC, ET0/RC, DI0/RC, ABS/CS, TR0/CS, ET0/CS, DI0/CS, FV/FM and PI, respectively. Plant survival on the x-axis (0-100) is a number of surviving (regrowing) plants examined after three weeks of regrowth in glasshouse (temp. about +15°C) and expressed as a percentage of plant survival. (DOCX 1369 KB) [file 13353_2021_660_MOESM4_ESM.docx]

A

J

H

F

D

I

G

E

C

B

**Figure S2.** Mean values of chlorophyll *a* fluorescence parameters measured in DH population after cold-acclimation in three different field conditions. A, B, C, D, E, F, G, H, I, J charts were made for ABS/RC, TR_0_/RC, ET_0_/RC, DI_0_/RC, ABS/CS, TR_0_/CS, ET_0_/CS, DI_0_/CS, F_V_/F_M_ and PI, respectively. Plant survival on the x-axis (0-100) is a number of surviving (regrowing) plants examined after three weeks of regrowth in glasshouse (temp. about +15°C) and expressed as a percentage of plant survival.
