# Supplementary material for: Quantitative trait loci and candidate genes associated with freezing tolerance of winter triticale (× Triticosecale Wittmack)
Source: J Appl Genet. 2021 Sep 7;63(1):15–33. doi: 10.1007/s13353-021-00660-1 (PMC8755666; doi:10.1007/s13353-021-00660-1)
Supplement: Supplementary file 6 — Data on maximum expected number of recombinations per individual (Max_rec) extracted and sum of adjacent recombination frequencies (Mean_rec) from JoinMap. Percentage of lines without crossing-over (c-o) was calculated in Excel. (DOCX 19 KB) [file 13353_2021_660_MOESM6_ESM.docx]

**Table S2. Data on maximum expected number of recombinations per individual (Max_rec) extracted and sum of adjacent recombination frequencies (Mean_rec) from JoinMap.** Percentage of lines without crossing-over (c-o) was calculated in Excel.

| **Linkage group** | **Max_rec** | **Mean_rec** | **Lines without c-o [%]** |
| --- | --- | --- | --- |
| 1A | 3 | 0.531 | 58.7 |
| 2A | 1.1 | 0.419 | 58.7 |
| 3A | 2 | 0.396 | 65.2 |
| 4A | 3 | 0.748 | 50.0 |
| 5A | 3 | 0.763 | 38.0 |
| 6A | 2 | 0.463 | 59.8 |
| 7A.1 | 3 | 0.317 | 58.7 |
| 7A.2 | 2 | 0.524 | 52.2 |
| 1B | 6 | 0.802 | 53.3 |
| 2B.1 | 2 | 0.627 | 46.7 |
| 2B.2 | 3 | 0.485 | 68.5 |
| 3B.1 | 2 | 0.228 | 83.7 |
| 3B.2 | 4 | 1.009 | 45.7 |
| 4B | 2 | 0.415 | 60.9 |
| 5B | 2 | 0.406 | 73.9 |
| 6B | 3 | 0.928 | 31.5 |
| 7B | 7 | 0.866 | 45.7 |
| 1R | 2 | 0.143 | 87.0 |
| 3R | 3 | 0.306 | 73.9 |
| 4R | 4 | 0.698 | 54.3 |
| 5R | 3 | 0.366 | 77.2 |
| 6R | 3* | 1.012 | 31.5 |

*- line nr 12 was excluded (as it showed 14 recombination events that is unlikely to be result of c-o only)
