# Supplementary material for: Quantitative trait loci and candidate genes associated with freezing tolerance of winter triticale (× Triticosecale Wittmack)
Source: J Appl Genet. 2021 Sep 7;63(1):15–33. doi: 10.1007/s13353-021-00660-1 (PMC8755666; doi:10.1007/s13353-021-00660-1)
Supplement: Supplementary file 7 — Summary of QTL identified using SMA method for plants recovery REC (percentage of survived plants after freezing) after freezing test performed during winter 2011/2012 (Experiment 1) and 2012/2013 (Experiments 2, 3).Table S4. Summary of QTL for electrolyte leakage (EL) from leaves after freezing identified using SMA method. Table S5. Summary of QTL for Fv/Fm (the yield of the energy trapping in PSII) and PI (performance indexes of PSII) identified using SMA method. QTL name includes each parameter name (after the letter Q). Table S6. Summary of QTL for energy fluxes (per single, active PSII reaction center; RC) identified using SMA method. QTL name includes each parameter name (after the letter Q). Abbreviations: ABS/RC - absorbed energy flux per the single, active PSII reaction center (RC); TR0/RC - trapped energy flux in PSII reaction centers per the single, active PSII reaction center; DI0/RC - dissipation of energy in PSII reaction centers per the single, active PSII reaction center. Table S7. Summary of QTL for phenomenological energy fluxes (per leaf cross-section; CS) identified using SMA method. QTL name includes each parameter name (after the letter Q). Abbreviations: ABS/CS - absorbed energy flux per leaf cross-section (CS); Tr0/CS - trapped energy flux in PSII reaction centers per leaf cross-section; ET0/CS - the energy flux for electron transport per leaf cross-section; DI0/CS – dissipation of energy in PSII reaction centers per leaf cross-section. (DOC 227 KB) [file 13353_2021_660_MOESM7_ESM.doc]

**Table S3 Summary of QTL identified using SMA method for plants recovery REC (percentage of survived plants after freezing) after freezing test performed during winter 2012/2013 (Experiment 1) and 2013/2014 (Experiments 2, 3)**.

| **QTL** | **Experiment No.** | **Flanking markers** | **Interval/Position** | **-2ln(L0/L1) ^a^** | **R^2^ (%) ^b^** | **Add ^c^** | **Favorable allele ^d^** |
| --- | --- | --- | --- | --- | --- | --- | --- |
| *Qrec.hm-4A.1* | 1 | *wPt-0162-wPt-7280* | 0.0-32.3 | 6.6 | 5.8 | 4.4 | H |
|  | 2 | *wPt-6728(4)-wPt-7280* | 21.1-32.3 | 5.4 | 5.8 | 6.0 | H |
|  | 3 | *wPt-6728(4)-wPt-7280* | 21.1-32.3 | 5.7 | 6.8 | 5.4 | H |
| *Qrec.hm-7A-1.1** | 1 | *wPt-3523-wPt-6824(2)* | 43.8-57.2 | 5.1 | 7.4 | 4.8 | H |
|  | 2 | *wPt-6668(4)-wPt-2056* | 27.5-61.8 | 9.1 | 7.0 | 5.8 | H |
|  | 3 | *wPt-6668(4)-wPt-2056* | 27.5-61.8 | 10.4 | 7.7 | 4.4 | H |
| *Qrec.hm-7A-2.1* | 1 | *wPt-6620(3)-tPt-513668* | 47.9-53.3 | 8.6 | 7.3 | 6.1 | H |
|  | 2 | *wPt-6620(3)-tPt-513668* | 47.9-53.3 | 5.9 | 5.5 | 6.8 | H |
|  | 3 | *wPt-6620(3)-tPt-513668* | 47.9-53.3 | 5.4 | 5.1 | 4.6 | H |
| *Qrec.hm-1B.1** | 1 | *wPt-5899(3)-wPt-6240* | 79.9-93.3 | 7.9 | 6.0 | 2.8 | H |
|  | 2 | *wPt-5675(8)-wPt-6240* | 54.6-93.3 | 14.6 | 10.2 | 6.2 | H |
|  | 3 | *wPt-5675(8)-wPt-6240* | 79.9-93.3 | 9.4 | 7.3 | 5.2 | H |
| *Qrec.hm-2B-1.1** | 1 | *wPt-9402(4)-wPt-1920* | 36.3-46.2 | 5.3 | 5.9 | 3.9 | H |
|  | 2 | *tPt-4627-wPt-1920* | 21.9-46.2 | 25.5 | 15.8 | 6.1 | H |
|  | 3 | *wPt-9402(4)-wPt-1920* | 36.3-46.2 | 14.1 | 12.2 | 7.3 | H |
| *Qrec.hm-7B.1* | 1 | *wPt-9467-wPt-2273(2)* | 16.7-30.0 | 6.2 | 6.1 | 3.4 | H |
|  | 2 | *wPt-9467-wPt-2273(2)* | 16.7-30.0 | 15.1 | 8.5 | 5.6 | H |
|  | 3 | *wPt-9467-wPt-2273(2)* | 16.7-30.0 | 11.4 | 7.3 | 5.3 | H |
| *Qrec.hm-4R.1** | 1 | *tPt-505225(7)-wPt-4487(3)* | 13.2-33.9 | 10.2 | 7.6 | -4.4 | M |
| *Qrec.hm-4R.2** | 1 | *tPt-402443(4)-tPt-508131* | 57.2-69.4 | 6.7 | 7.3 | -4.1 | M |
|  | 3 | *tPt-402443(4)-tPt-508131* | 57.2-69.4 | 5.6 | 4.9 | -4.5 | M |
| *Qrec.hm-4R.3* | 3 | *tPt-401048(4)* | 71.6 | 5.2 | 4.4 | -4.5 | M |
| *Qrec.hm-5R.2* | 2 | *rPt-505151-rPt-507354(4)* | 6.3-8.0 | 5.2 | 5.1 | -5.9 | M |
| *Qrec.hm-5R.1** | 2 | *rPt-505490(14)-tPt-508190* | 16.8-28.7 | 8.4 | 5.8 | -5.7 | M |

^a^ -2ln(L0/L1) - probability ratio for the SMA analysis; ^b^ R^2^ (%) – the percentage of phenotypic variance explained by the QTL; ^c^ Add - additive effects of QTL expressed in the trait unit; ^d^ Favorable allele for each QTL: H - cv. Hewo and M - cv. Magnat; * - QTL confirmed with Composite Interval Mapping.

**Table S4. Summary of QTL for electrolyte leakage (EL) from leaves after freezing identified using SMA method.**

| **QTL** | **Experiment No.** | **Flanking markers** | **Interval/Position** | **-2ln(L0/L1) ^a^** | **R^2^ (%) ^b^** | **Add ^c^** | **Favorable allele ^d^** |
| --- | --- | --- | --- | --- | --- | --- | --- |
| *Qel.hm-4A.1* | 2 | *wPt-6728(4)-wPt-7280* | 21.1-32.3 | 5.2 | 3.3 | 5.4 | H |
|  | 3 | *wPt-6728(4)-wPt-7280* | 21.1-32.3 | 5.1 | 6.4 | 4.2 | H |
| *Qel.hm-7A-1.1** | 3 | *wPt-6668(4)* | 27.5 | 5.2 | 5.8 | -4.0 | M |
| *Qel.hm-7A-1.2** | 3 | *wPt-8418-wPt-9207(2)* | 30.4-38.3 | 6.1 | 4.8 | -3.7 | M |
| *Qel.hm-7A-2.1** | 2 | *wPt-1436-tPt-2230(2)* | 0.0-19.9 | 5.6 | 3.4 | 3.4 | H |
|  | 3 | *wPt-1436-tPt-2230(2)* | 0.0-19.9 | 8.4 | 5.7 | 3.9 | H |
| *Qel.hm-4B.1* | 2 | *gwm375-wPt-7412(2)* | 4.4-16.4 | 8.3 | 6.4 | 5.4 | H |
|  | 3 | *gwm375-wPt-8543* | 4.4-10.9 | 12.6 | 11.8 | 4.5 | H |
| *Qel.hm-4R.1** | 2 | *rPt-389466-rPt-389872(5)* | 9.8-16.5 | 22.8 | 19.6 | 9.8 | H |
|  | 3 | *rPt-389466-rPt-389872(5)* | 9.8-16.5 | 12.9 | 12.8 | 4.9 | H |
| *Qel.hm-4R.2* | 2 | *tPt-513729-wPt-4487(3)* | 32.9-45.0 | 17.0 | 13.3 | 7.8 | H |
|  | 3 | *tPt-513729-wPt-4487(3)* | 32.9-45.0 | 8.5 | 7.8 | 4.5 | H |
| *Qel.hm-5R.3* | 2 | *rPt-505151-gwm136* | 0.0-6.3 | 5.6 | 5.9 | 5.5 | H |
|  | 3 | *rPt-505151-gwm136* | 0.0-6.3 | 6.0 | 4.2 | 4.2 | H |
| *Qel.hm-5R.2* | 3 | *rPt-505590-tPt-401845(4)* | 20.0-24.4 | 5.3 | 4.8 | 3.8 | H |
| *Qel.hm-5R.1** | 3 | *rPt-506350(10)-wmc289* | 32.0-32.9 | 6.6 | 6.9 | 4.9 | H |

^a^ -2ln(L0/L1) - probability ratio for the SMA analysis; ^b^ R^2^ (%) – the percentage of phenotypic variance explained by the QTL; ^c^ Add - additive effects of QTL expressed in the trait unit; ^d^ Favorable allele for each QTL: H - cv. Hewo and M - cv. Magnat; * - QTL confirmed with Composite Interval Mapping.

**Table S5. Summary of QTL for *F_v_/F_m_* (the yield of the energy trapping in PSII) and PI (performance indexes of PSII) identified using SMA method. QTL name includes each parameter name (after the letter Q).**

| **QTL** | **Experiment no** | **Flanking markers** | **Interval/Position** | **-2ln(L0/L1) ^a^** | **R^2^ (%) ^b^** | **Add ^c^** | **Favorable allele ^d^** |
| --- | --- | --- | --- | --- | --- | --- | --- |
| *Qfvfm.hm-5R.1* | 3 | *wmc289-rPt-390144* | 32.9-36.6 | 5.9 | 5.3 | -0.4 | M |
|  | 3 | *rPt-507354(4)-tPt-508190* | 8.0-28.7 | 6.1 | 5.9 | -0.4 | M |
| *Qpi.hm-4A.1* | 1 | *wPt-0162-wPt-2151* | 0.0-56.6 | 7.2 | 7.7 | 0.7 | H |
|  | 3 | *wPt-1091-wPt-7280* | 21.1-32.3 | 8.4 | 6.2 | 0.4 | H |
| *Qpi.hm-5A.2** | 1 | *wmc327.1-wPt-1370(2)* | 0.0-19.4 | 9.9 | 7.9 | 0.7 | H |

^a^ -2ln(L0/L1) - probability ratio for the SMA analysis; ^b^ R^2^ (%) – the percentage of phenotypic variance explained by the QTL; ^c^ Add - additive effects of QTL expressed in the trait unit; ^d^ Favorable allele for each QTL: H - cv. Hewo and M - cv. Magnat; * - QTL confirmed with Composite Interval Mapping.

**Table S6. Summary of QTL for energy fluxes (per single, active PSII reaction centre; RC) identified using SMA method.** QTL name includes each parameter name (after the letter Q). Abbreviations: ABS/RC - absorbed energy flux per the single, active PSII reaction centre (RC); TR_0_/RC - trapped energy flux in PSII reaction centers per the single, active PSII reaction centre; DI_0_/RC - dissipation of energy in PSII reaction centers per the single, active PSII reaction centre.

| **QTL** | **Trait** | **Experiment No.** | **Flanking markers** | **Interval/Position** | **-2ln(L0/L1) ^a^** | **R^2^ (%) ^b^** | **Add ^c^** | **Favorable allele ^d^** |
| --- | --- | --- | --- | --- | --- | --- | --- | --- |
| *Qabsrc.hm-4A.1* | ABS/RC | 3 | *wPt-0162-wPt-6728(4)* | 0.0-21.1 | 6.0 | 5.9 | -2.5 | M |
| *Qabsrc.hm-5A.2* | ABS/RC | 2 | *wmc327.1-wPt-3334(2)* | 0.0-31.7 | 11.5 | 10.3 | -0.1 | M |
|  |  | 3 | *wmc327.1-wPt-1370* | 0.0-19.4 | 8.6 | 7.9 | -2.9 | M |
| *Qabsrc.hm-5B.1* | ABS/RC | 2 | *gwm335-wmc537* | 18.6-41.9 | 13.7 | 9.3 | -0.1 | M |
|  |  | 3 | *gwm499-wmc537* | 38.5-41.9 | 5.3 | 4.9 | -2.4 | M |
| *Qtrorc.hm-.4R.1* | TR_0_/RC | 3 | *rPt-389872(5)* | 16.5 | 5.2 | 5.0 | -0.1 | M |
| *Qtrorc.hm-.4R.2* | TR_0_/RC | 3 | *tPt-508199(2)-wPt-4487(3)* | 33.9-45.0 | 5.7 | 5.4 | -0.1 | M |
| *Qdiorc.hm-4R.1* | DI_0_//RC | 3 | *rPt-389872(5)* | 16.5 | 5.6 | 6.1 | -0.3 | M |
| *Qdiorc.hm-4R.2* | DI_0_//RC | 3 | *tPt-513729-wPt-4487(3)* | 32.9-45.0 | 6.2 | 5.8 | -0.4 | M |

^a^ -2ln(L0/L1) - probability ratio for the SMA analysis; ^b^ R^2^ (%) – the percentage of phenotypic variance explained by the QTL; ^c^ Add - additive effects of QTL expressed in the trait unit; ^d^ Favorable allele for each QTL: H - cv. Hewo and M - cv. Magnat; * - QTL confirmed with Composite Interval Mapping.

**Table S7. Summary of QTL for phenomenological energy fluxes (per leaf cross-section; CS) identified using SMA method.** QTL name includes each parameter name (after the letter Q). Abbreviations: ABS/CS - absorbed energy flux per leaf cross-section (CS); Tr_0_/CS - trapped energy flux in PSII reaction centers per leaf cross-section; ET_0_/CS - the energy flux for electron transport per leaf cross-section; DI_0_/CS – dissipation of energy in PSII reaction centers per leaf cross-section.

| **QTL** | **Trait** | **Experiment no** | **Flanking markers** | **Interval/Position** | **-2ln(L0/L1) ^a^** | **R^2^ (%) ^b^** | **Add ^c^** | **Favorable allele ^d^** |
| --- | --- | --- | --- | --- | --- | --- | --- | --- |
| *Qabscs.hm-4A.1* | ABS/CS | 3 | *wPt-6728(4)* | 21.1 | 5.1 | 5.3 | 16.9 | H |
| *Qabscs.hm-5A.1* | ABS/CS | 2 | *wPt-1052-gwm126(2)* | 45.2-51.8 | 6.7 | 6.0 | -10.9 | M |
| *Qabscs.hm-5A.2** | ABS/CS | 3 | *wmc327.1-wPt-1370* | 0.0-19.4 | 6.4 | 6.8 | 18.5 | H |
| *Qabscs.hm-5B.1** | ABS/CS | 1 | *gdm147(2)-wmc537* | 15.3-41.9 | 8.4 | 7.0 | -7.2 | M |
|  |  | 2 | *wPt-5514-wmc537* | 35.2-41.9 | 7.6 | 7.6 | -11.5 | M |
|  |  | 3 | *wPt-7167-wmc537* | 28.6-41.9 | 12.7 | 9.3 | 25.9 | H |
| *Qabscs.hm-5B.2* | ABS/CS | 2 | *wPt-5120(2)-wPt-4996(4)* | 0.0-6.6 | 6.5 | 7.1 | -11.4 | M |
|  |  | 3 | *wPt-5120(2)-wPt-4996(4)* | 0.0-6.6 | 5.3 | 3.2 | 15.9 | H |
| *Qabscs.hm-4R.1* | ABS/CS | 1 | *tPt-398700(2)* | 0.0 | 6.9 | 6.7 | -6.5 | M |
|  |  | 1 | *tPt-402470-rPt-402563(5)* | 22.6-25.2 | 5.3 | 5.4 | -5.7 | M |
|  |  | 3 | *tPt-398700(2)-tPt-411422(14)* | 0.0-11.0 | 6.6 | 5.1 | -18.7 | M |
| *Qabscs.hm-4R.2** | ABS/CS | 2 | *rPt-389466-wPt-4487* | 9.8-45.0 | 10.4 | 8.8 | -13.2 | M |
|  |  | 3 | *tPt-402470-wPt-4487* | 22.6-45.0 | 6.7 | 6.2 | -18.8 | M |
| *Qabscs.hm-4R.3* | ABS/CS | 1 | *508131-tPt-401048(4)* | 69.4-71.6 | 10.8 | 10.2 | -8.0 | M |
| *Qabscs.hm-7A-1.1* | ABS/CS | 3 | *wPt-6668(4)-wPt-2056* | 27.5-61.8 | 10.3 | 6.2 | 23.0 | H |
| *Qabscs.hm-5R.1** | ABS/CS | 2 | *tPt-390596(4)-tPt-508190(6)* | 22.2-28.7 | 6.3 | 5.7 | -11.4 | M |
|  |  | 3 | *tPt-508190(6)-tPt-399743(3)* | 28.7-34.2 | 5.9 | 5.8 | -18.9 | M |
| *Qetocs.hm-4A.1** | ET_0_/CS | 1 | *wPt-7280* | 32.3 | 8.5 | 10.0 | 4.2 | H |
|  |  | 3 | *wPt-6728(4)-wPt-7280* | 21.1-32.3 | 8.2 | 8.7 | 9.1 | H |
| *Qetocs.hm-5A.2** | ET_0_/CS | 2 | *wmc327.1-wPt-1370* | 0.0-19.4 | 6.4 | 6.6 | 4.9 | H |
|  |  | 3 | *wmc327.1-wPt-1370* | 0.0-19.4 | 7.5 | 6.7 | 8.5 | H |
| *Qetocs.hm-5B.1* | ET_0_/CS | 3 | *gwm499-wmc537* | 38.5-41.9 | 8.4 | 6.7 | 9.2 | H |
| *Qetocs.hm-5B.2* | ET_0_/CS | 1 | *wPt-5120(2)-gwm335* | 0.0-18.6 | 9.2 | 6.9 | -4.2 | M |
| *Qetocs.hm-5R.1* | ET_0_/CS | 2 | *gwm136-rPt-390144* | 0.0-36.6- | 21.6 | 16.4 | -9.1 | M |
|  |  | 3 | *tPt-399743(3)* | 34.2 | 5.2 | 4.4 | -6.9 | M |
|  |  | 3 | *tPt-508190(6)* | 28.7 | 5.6 | 5.1 | -7.4 | M |
|  |  | 3 | *tPt-508895(2)-tPt-401845(4)* | 23.3-24.4 | 5.1 | 4.0 | -6.8 | M |
|  |  | 3 | *rPt-507354(4)-rPt-505590* | 8.0-20.0- | 6.0 | 5.4 | -8.0 | M |
| *Qtrocs.hm-4A.1* | TR_0_/CS | 3 | *wPt-5857-wPt-7280* | 16.7-32.3 | 10.8 | 9.7 | 27.8 | H |
| *Qtrocs.hm-5A.1* | TR_0_/CS | 2 | *wPt-1052-wPt-3334(2)* | 45.2-46.4 | 5.4 | 5.6 | -7.3 | M |
| *Qtrocs.hm-5A.2** | TR_0_/CS | 3 | *wmc327.1-wPt-1370* | 0.0-19.4 | 10.1 | 9.1 | 26.5 | H |
| *Qtrocs.hm-5B.1* | TR_0_/CS | 1 | *gdm147(2)-wmc537* | 15.3-41.9 | 10.0 | 7.9 | -5.0 | M |
|  |  | 2 | *wPt-5514-wmc537* | 35.2-41.9 | 7.5 | 7.2 | -8.6 | M |
|  |  | 3 | *wPt-5514-wmc537* | 35.2-41.9 | 10.2 | 7.0 | -2.3 | M |
| *Qtrocs.hm-5B.2* | TR_0_/CS | 2 | *wPt-4996(4)-wPt-5120(2)* | 0.0-6.6 | 5.1 | 4.9 | -6.6 | M |
| *Qtrocs.hm-4R.1* | TR_0_/CS | 1 | *tPt-402470-rPt-402563(5)* | 22.6-25.2 | 5.8 | 6.2 | -3.7 | M |
|  |  | 2 | *rPt-389466-tPt-402470* | 9.8-22.6 | 8.4 | 7.1 | -8.9 | M |
| *Qtrocs.hm-4R.2** | TR_0_/CS | 2 | *tPt-513729-wPt-4487(3)* | 32.9-45.0 | 7.6 | 6.7 | -8.4 | M |
| *Qtrocs.hm-4R.3* | TR_0_/CS | 1 | *tPt-508131-tPt-4011048(4)* | 69.4-71.6 | 7.0 | 6.8 | -4.1 | M |
| *Qtrocs.hm-5R.1** | TR_0_/CS | 1 | *rPt-505449(6)* | 13.5 | 5.5 | 4.7 | -3.4 | M |
|  |  | 2 | *gwm136-rPt-390144* | 0.0-36.6 | 11.2 | 7.2 | -10.9 | M |
|  |  | 3 | *wmc289-rPt-390144* | 32.9-36.6 | 6.9 | 6.4 | -23.7 | M |
|  |  | 3 | *tPt-508190(6)* | 28.7 | 6.1 | 6.7 | -22.9 | M |
|  |  | 3 | *rPt-507354(4)-tPt-401845(4)* | 8.0-24.4 | 6.7 | 5.8 | -22.8 | M |
| *Qdiocs.hm-4A.1* | DI_0_/CS | 1 | *wPt-0162-wPt-6728(4)* | 0.0-21.1 | 7.2 | 7.2 | -2.5 | M |
|  |  | 2 | *wPt-0162* | 0.0 | 5.5 | 4.1 | -2.3 | M |
|  |  | 3 | *wPt-7280(4)* | 21.1 | 5.6 | 6.5 | -17.3 | M |
| *Qdiocs.hm-4R.1* | DI_0_/CS | 3 | *rPt-389466-rPt-389872(5)* | 9.8-16.5 | 9.8 | 8.3 | -21.5 | M |
| *Qdiocs.hm-4R.2* | DI_0_/CS | 3 | *tPt-513729-wPt-4487(3)* | 32.9-45.0 | 7.9 | 7.9 | -19.4 | M |
| *Qdiocs.hm-4R.3* | DI_0_/CS | 1 | *tPt-508131-tPt-401048(4)* | 69.4-71.6 | 10.8 | 9.8 | -2.9 | M |
| *Qdiocs.hm-7A-1.4* | DI_0_/CS | 2 | *wPt-6184-wPt-3523* | 29.3-43.8 | 7.3 | 7.0 | 2.9 | H |
| *Qdiocs.hm-5A.1* | DI_0_/CS | 2 | *wPt-1052-gwm126(2)* | 45.2-51.8 | 5.3 | 4.7 | -2.5 | M |
| *Qdiocs.hm-5A.2* | DI_0_/CS | 1 | *wmc327.1-wPt-5250* | 0.0-35.7 | 7.6 | 6.0 | -2.5 | M |
| *Qdiocs.hm-5B.1* | DI_0_/CS | 1 | *gwm499-wmc537* | 38.5-41.9- | 5.9 | 5.1 | -2.0 | M |
|  |  | 2 | *wPt-5514-wmc537* | 35.2-41.9- | 7.6 | 7.5 | -3.0 | M |
| *Qdiocs.hm-5B.2* | DI_0_/CS | 2 | *wPt-5120(2)-wPt-4996(4)* | 0.0-6.6 | 9.3 | 8.7 | -3.5 | M |

^a^ -2ln(L0/L1) - probability ratio for the SMA analysis; ^b^ R^2^ (%) – the percentage of phenotypic variance explained by the QTL; ^c^ Add - additive effects of QTL expressed in the trait unit; ^d^ Favorable allele for each QTL: H - cv. Hewo and M - cv. Magnat; * - QTL confirmed with Composite Interval Mapping.
